# Neuroprotective Effects of Transcranial Pulsed Current Stimulation: Modulation of Microglial Polarization in Traumatic Brain Injury

**DOI:** 10.1111/cns.70606

**Published:** 2025-09-07

**Authors:** Peng Yao, Bingkai Ren, Qianhui Zhou, Yang Bai, Zhen Feng

**Affiliations:** ^1^ Affiliated Rehabilitation Hospital Jiang Xi Medical College, Nanchang University Nanchang Jiangxi China; ^2^ Rehabilitation Medicine Clinical Research Center of Jiangxi Province Nanchang Jiangxi China; ^3^ Key Laboratory of Jiangxi Provincial Health Commission for DOC Rehabilitation Nanchang Jiangxi China; ^4^ The First Affiliated Hospital of Nanchang University, Jiang Xi Medical College, Nanchang University Nanchang Jiangxi China

**Keywords:** microglia, neuroplasticity, NF‐κB pathway, orexin‐A, transcranial pulsed current stimulation, traumatic brain injury

## Abstract

**Objective:**

Traumatic brain injury (TBI), a prevalent neurological disorder worldwide, is marked by varying degrees of neurological dysfunction. A key contributor to secondary damage and impediments in the repair process is the unregulated activation of microglia, which triggers neuroinflammation. Emerging evidence highlights the therapeutic potential of transcranial pulsed current stimulation (tPCS) in mitigating neurological deficits. However, despite these promising neuroprotective effects, its role and exact mechanisms in TBI remain unclear.

**Methods:**

Herein, a mouse model of TBI was established, and daily 30‐min tPCS treatments were administered for five consecutive days. Subsequently, we conducted comprehensive assessments of neurological function, microglial activation status, and neuroplasticity in the treated subjects. Additionally, a co‐culture system of BV2 and HT22 cells was developed, using LPS to activate microglia, to explore potential neuroprotective mechanisms.

**Results:**

Our findings revealed that tPCS plays a crucial role in mitigating neuroinflammation and promoting neurological recovery following TBI. The underlying mechanism likely involves tPCS enhancing orexin‐A (OX‐A) expression, which subsequently suppresses the NF‐κB pathway and promotes the expression of neurorepair‐related markers. In vitro experiments further clarified these findings, demonstrating that OX‐A effectively inhibited LPS‐induced M1 microglial polarization and promoted a shift towards the M2 phenotype. Furthermore, OX‐A significantly reduced intracellular ROS production and microglia‐induced neuronal apoptosis.

**Conclusion:**

These findings indicate that tPCS regulates microglial phenotype via the OX‐A/NF‐κB pathway, thereby suppressing neuroinflammation and enhancing neuroplasticity. These results provide a new perspective for the rehabilitation of individuals with TBI.

## Graphical abstract

The schematic diagram illustrates the neuroprotective effects of tPCS on TBI. Treatment with tPCS mitigates neuroinflammation and promotes neuroregeneration by targeting the OX‐A/OX1R‐mediated NF‐κB pathway, thereby facilitating the recovery of neurological function in TBI‐afflicted mice.

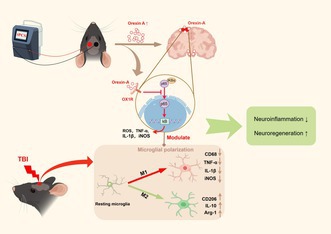

## 1 Introduction

Traumatic brain injury (TBI), a life‐threatening neuropathological condition, poses a significant public health challenge [1]. Epidemiological studies reveal that the incidence of TBI is substantial and rising, with approximately 2.5 million cases occurring annually in the United States, leading to over 280,000 hospitalizations and 50,000 deaths [2]. While TBI is initially triggered by traumatic events causing compression and tearing of brain tissue, secondary pathophysiological mechanisms, including immuno‐inflammatory response, mitochondrial dysfunction, and programmed cell death, often exacerbate the initial injury [3, 4]. Specifically, the resulting neuroinflammation can further amplify secondary injuries, impede tissue repair processes, ultimately resulting in brain tissue loss and neurological dysfunction [5]. Currently, effective treatments are lacking, underscoring the urgent need for more effective therapeutic strategies to improve patient outcomes.

Microglia, critical mediators of immune‐inflammatory responses, are essential for limiting tissue damage and promoting neural repair [6]. Following acute brain injuries like ischemic stroke [7] and TBI [8], both classically activated (M1) and alternatively activated (M2) microglia are initially upregulated in the peri‐lesional cortex. However, under intense stimulation or sustained stimulation, microglia tend to polarize towards the M1 phenotype rather than the M2 [9, 10]. This shift is characterized by increased secretion of pro‐inflammatory cytokines such as TNF‐α and IL‐1β, and decreased production of immunoregulatory cytokines including TGF‐β and IL‐10 [11]. The excessive accumulation of pro‐inflammatory mediators can lead to overproduction of reactive oxygen species [12], disrupting mitochondrial membrane potential and ultimately resulting in uncontrolled inflammatory cascades, apoptosis, and impaired neurorepair. Strategies that suppress M1 microglial polarization or promote M2 polarization can alleviate neuroinflammation and secondary damage [13]. Therefore, restoring microglial phenotype homeostasis offers a promising approach to promote neurological recovery following TBI.

Neuromodulation techniques, such as transcranial electrical stimulation and deep brain stimulation, are increasingly utilized to promote neurological recovery following brain injury and stroke [14]. Transcranial pulsed current stimulation (tPCS) is a novel and noninvasive technique that modulates cortical excitability by delivering pulsed currents to the cerebral cortex, thereby influencing neurological function [15, 16]. Computational modeling reveals that tPCS‐generated electric fields are capable of permeating the cerebral cortex and reaching subcortical regions, effectively achieving deep neuromodulation [17]. Accumulating evidence supports tPCS's capacity to modulate immune‐inflammatory responses and promote neurological recovery. For example, tPCS targeting the primary motor or sensory cortex has been shown to enhance cortical excitability [18], improve performance in attention‐switching tasks [19], increase efficiency in complex mathematical processing [20], and facilitate motor functional recovery in patients with Parkinson's disease (PD) [21]. Preclinical studies further suggest that tPCS can ameliorate poststroke neurological dysfunction by modulating glial cell calcium responses [22], inhibiting neuroinflammation and pyroptosis, and upregulating neurotrophic factor expression [23, 24]. Despite accumulating evidence indicating a significant neuroprotective role for tPCS, its specific effects and mechanisms in TBI remain poorly understood.

Orexin‐A (OX‐A), a neuropeptide synthesized and secreted by the lateral hypothalamus, modulates physiological processes, including sleep/wake cycles, feeding behavior, and injury repair by binding to and activating orexin receptor type 1 and type 2 (OX1R and OX2R) [25]. Studies have demonstrated that exogenous OX‐A can attenuate neuroinflammation and enhance neurological function following ischemic stroke [26]. Furthermore, reduced OX‐A expression has been correlated with the severity and poor prognosis of subarachnoid hemorrhage [27, 28]. These findings are supported by studies employing orexin/ataxin‐3 transgenic mice, which demonstrate that OX‐A can reverse stroke‐induced M1 microglial polarization and neurological deficits [29]. Furthermore, our previous investigations indicated that stimulating the lateral hypothalamus increases OX‐A secretion, subsequently elevating OX‐A levels in the prefrontal cortex and hippocampus [30, 31]. In vitro experiments further revealed that OX‐A can mitigate LPS‐induced inflammatory damage while promoting neuronal proliferation and migration of neural stem cells [32]. Based on these findings, we hypothesize that tPCS may restore the homeostasis of microglia by promoting OX‐A release, thereby alleviating neurological dysfunction following TBI.

In this study, we aimed to investigate the therapeutic effects of tPCS on neuroinflammation and neurological recovery in mice with TBI, as well as to explore the potential mechanisms by which OX‐A regulates microglial polarization.

## 2 Materials and Methods

### 2.1 Animals and Group

Preclinical and clinical evidence suggests that female hormones, including estrogen and progesterone, can influence steroid hormone synthesis, electrolyte balance, and neurotrophic factor expression [33, 34]. Consequently, female subjects may exhibit greater resilience to brain injury, potentially leading to accelerated neurological recovery and improved overall outcomes compared to males [35, 36]. To avoid potential confounding effects of sex or hormonal factors that might diminish or counteract the neuromodulatory effects of tPCS on neurorepair and behavioral outcomes, this study included only male subjects.

Healthy male C57BL/6 murine specimens, which were 8 weeks and weighing 25–30 g, were procured from Zhejiang Vital River Laboratory Animal Technology Co. Ltd. for utilization as experimental subjects. Animals were accommodated within a controlled milieu exhibiting a 12‐h photoperiod with unrestricted access to food and water. This study was approved by the Animal Care and Use Committee of Nanchang University (ID: CDYFY‐IACUC‐202302QR058).

Animal experiments were conducted in two phases. In the preliminary phase, 24 mice (27 mice were used, with three mortalities) were randomly attributed into three cohorts: a sham‐operated group (Sham, *n* = 8), a TBI operation group (TBI, *n* = 8, with two mortalities), and a TBI group receiving tPCS stimulation (tPCS, *n* = 8, with one mortality). This design aims to assess the impact of tPCS on neurological recovery post‐TBI. Subsequently, in the second experimental phase, 72 mice (77 mice were used, with five mortalities) were randomly allocated into four cohorts: a Sham group (*n* = 18), a TBI group (*n* = 18, with two mortalities), a tPCS group (*n* = 18, with two mortalities), and a tPCS group receiving SB334867 treatment group (tPCS+SB334867, *n* = 18, with one mortality), to further investigate the potential mechanisms through which tPCS promotes neurological recovery post‐TBI.

### 2.2 TBI Operation

In this study, we induced TBI in mice using the controlled cortical impact (CCI) procedure, as previously described [37]. Briefly, mice were anesthetized with an intraperitoneal injection of 5% sodium pentobarbital. Following disinfection, a midline scalp incision was made, and a 5.0 mm diameter craniotomy was created over the left parietal cortex (centered at AP: −0.5 mm, ML: −2.5 mm). A CCI device (RWD Life Sciences) was employed to induce moderate‐to‐severe TBI with the following parameters: impact depth 2.0 mm, dwell time 100 ms, and impact velocity 4.0 m/s. Following the impact, the craniotomy was closed, and the scalp incision was sutured. Sham‐operated animals underwent the same surgical procedures, excluding the cortical impact. Postoperatively, mice received a subcutaneous injection of 0.1 mg/kg buprenorphine for analgesia and were placed on a heating pad until they regained consciousness.

### 2.3 tPCS

Building upon established safety parameters for noninvasive transcranial electrical stimulation in small animal models, which generally limit current intensity to 2.0 mA [38] and single stimulation durations to 30 min [39]. Prior research has validated the safety of various stimulation frequencies, including 2 Hz, 5 Hz, 10 Hz, 20 Hz, and 50 Hz [40]. Notably, low‐frequency tPCS (1–20 Hz) combined with short pulse widths (1 ms, 2 ms, or 5 ms) has demonstrated the ability to modulate neuroplasticity [22] and exert neuroprotective effects [24, 31]. Given the potential for intracranial pressure fluctuations, the presence of cerebral edema, the modulatory effects of anesthetic agents on neural activity, and other potential risks to subjects following brain injury [41], this study initiated a 5‐day tPCS treatment protocol 72 h post‐TBI. This protocol consisted of daily 30‐min sessions of tPCS, employing a biphasic pulse with a frequency of 20 Hz, a current intensity of 1.0 mA, and a pulse width of 2 ms.

A programmable electrical stimulation device (Jingyi Medical Company, China) was employed to deliver tPCS. Briefly, mice were initially anesthetized with 3.5% isoflurane and maintained at 2.0% isoflurane anesthesia throughout the procedure. Subsequently, the mice were secured in a stereotaxic frame. The tPCS anode was positioned over the injured cortical region, while the cathode was placed on the corresponding contralateral side. Mice in the Sham group received 30 min of isoflurane anesthesia without additional intervention. During tPCS administration, vital signs of the mice were continuously monitored using an animal multiparameter monitoring system.

### 2.4 The Modified Neurological Severity Score (mNSS)

The mNSS was employed to assess overall neurological deficits following TBI [37]. Two researchers, blinded to the experimental groups, conducted the mNSS evaluations.

### 2.5 Drug Administration

Bromodeoxyuridine (BrdU, Solarbio, China) was dissolved and diluted in 0.9% saline to a concentration of 10 mg/mL. Mice received daily intraperitoneal injections of BrdU (50 mg/kg) from 1 to 7 days post‐operation (dpo). The selective OX1R inhibitor SB334867 (ab120164, Abcam) was dissolved in 5% DMSO and then diluted with 0.9% saline to a concentration of 10 mg/mL. The tPCS group received daily intraperitoneal injections of SB334867 (4 mg/kg) from 3 to 7 dpo, while the remaining cohorts received intraperitoneal injections of the vehicle for 5 days.

### 2.6 Neurological Behavior

To evaluate motor coordination and balance in TBI mice, the beam‐balance test and rotarod test were performed [42]. During the beam walking test's training phase, mice were placed at one end of a beam and stimulated with light to move towards a covered enclosure. In the subsequent testing phase, we recorded the time each mouse took to cross the beam. Similarly, the rotarod test included training and testing phases. During training, mice were placed on a rotating rod for 5 min to acclimate to the device with gradual deceleration. During the subsequent phase, the rod's speed gradually increased from 5 rpm to 30 rpm, and we recorded the latency to fall from the rotating rod for each mouse over 5 min.

To evaluate learning and memory in brain‐injured mice, we conducted Y‐maze, Morris water maze (MWM), and novel object recognition (NOR) tests following established protocols. In the Y‐maze and novel object recognition tests, a 5‐min training phase allowed mice to freely explore the environment. Subsequently, during a 5‐min testing phase, we quantified learning and memory by recording the time each mouse spent exploring the novel arm or novel object. The Morris water maze involved a 5‐day acquisition phase, during which mice learned to locate a hidden platform. On Day 6, the platform was removed, and mice underwent a probe trial where their swim paths were recorded over a 120‐s period to assess spatial memory retention.

### 2.7 Transcriptome Sequencing Analysis

At 7 dpo, mice from the TBI and tPCS treatment groups were euthanized via anesthetic overdose. Total RNA was extracted from the injured cortex, and cDNA libraries were constructed for transcriptome sequencing. BioDeep (Suzhou, China) performed the transcriptome sequencing. Differentially expressed genes (DEGs) between groups were identified using thresholds of |log2 Fold‐change| ≥ 1 and *p* < 0.1. R Studio was then used to visualize DEGs and perform Kyoto Encyclopedia of Genes and Genomes (KEGG) pathway enrichment analysis.

### 2.8 Evans Blue (EB) Staining

At 7 dpo, mice were injected via the tail vein with 2.0% EB dye. Two hours postinjection, animals were anesthetized and euthanized, followed by perfusion with 0.9% saline. Subsequently, brain tissues were excised, blotted dry, and weighed. To extract EB, brain samples were homogenized in DMSO for 24 h. Following centrifugation, the supernatant was collected, and its absorbance at 630 nm was measured with a multifunctional microplate reader.

### 2.9 Brain Water Content

At 7 dpo, mice were anesthetized and euthanized, and brain tissue samples were collected. Brains were bisected along the midline, and the wet weight was measured using an electronic balance. Specimens were then dried in an oven at 100°C for 48 h, and the dry weight was recorded. Brain water content is calculated as: [(Wet mass − Dry mass)/Wet mass] × 100%.

### 2.10 Nissl Staining and Hematoxylin–Eosin (HE) Staining

At 7 dpo, mice were anesthetized and euthanized, and their brains were extracted and fixed in 4% PFA for 24 h. Following embedding, 5 μm thick sections were prepared and stained with either HE or Nissl stain. At least three nonoverlapping regions of interest (ROIs) were randomly selected in the peri‐lesional cortex for image acquisition, followed by quantitative analysis utilizing ImageJ software. Lesion volume was quantified based on a previously established method [43].

### 2.11 Immunofluorescence (IF) Staining

For in vivo experiments, sections were subjected to boiling in citrate buffer for 10 min, naturally cooled, and then blocked with 3% BSA for 30 min. Subsequently, primary antibodies against OX1R (183701AP, Proteintech, China), ZO‐1 (ab307799, Abcam, USA), MAP2 (ab254264, Abcam), GAP43 (ab277627, Abcam), TGF‐β1 (ab215715, Abcam), IBA1 (ab178846, Abcam), Bcl‐xL (ab270253, Abcam), NeuN (ab104224, Abcam), CD31 (ab182981, Abcam), BrdU (Ab6326, Abcam), CD206 (#91992, Cell Signaling Technology), and CD86 (ab119857, Abcam) were used to the sections and incubated at 4°C for 12 h. The sections were then incubated with Alexa Fluor 488 or 555‐conjugated secondary antibodies for 2 h. Fluorescence microscopy was used to capture images from three randomly selected, nonoverlapping ROIs in the cortex. ImageJ software was then used to quantify positively stained cells or mean fluorescence intensity (MFI). In vitro, BV2 and HT22 cells were cocultured in a 6‐well plate system. Following coculture, cells underwent an IF staining protocol. Briefly, cells were fixed with 4% PFA, permeabilized with 0.5% Triton X‐100, and blocked with 5% BSA. Samples were then incubated with primary antibodies against specific markers at 4°C for 12 h, followed by secondary antibody labeling. Three random, nonoverlapping regions were captured per sample using a fluorescence microscope.

### 2.12 TdT‐Mediated dUTP Nick‐End Labeling (TUNEL) Staining

TUNEL staining was employed to assess neuronal death. After dehydration and dewaxing, sections were incubated with TUNEL working solution (Servicebio, China) at 37°C for 2 h, followed by DAPI staining at 4°C for 8 min. After thorough rinsing, the sections were dried, mounted, and examined under a fluorescence microscope. Images were captured using the fluorescence microscope, and ImageJ software was used to quantify positive cells.

### 2.13 Golgi Staining

Following anesthesia and euthanasia, whole brain tissues were extracted from mice and processed sequentially. Initially, the tissues were immersed in a Golgi staining solution (Servicebio), which was refreshed every 48 h for 2 weeks. Subsequently, the tissues underwent dehydration in a 15% sucrose solution at 4°C in darkness for 1.5 h. The solution was then replaced, and dehydration continued for 72 h. A vibratome (VT1000s, Leica) was used to section the tissues into 60 μm‐thick slices. These slices were then treated with a Golgi chromogen solution for 30 min, followed by thorough rinsing with ultrapure water. After complete drying, the slices were coverslipped and digitally scanned using a digital scanner (3DHISTECH, Hungary). Morphological analysis of dendritic spine density was performed using ImageJ software at 1000× magnification, quantifying the number of dendritic spines per 10 μm. Furthermore, dendritic complexity was assessed by measuring the number of intersections between dendrites and concentric circles.

### 2.14 Transmission Electron Microscopy (TEM)

Mitochondrial and synaptic structures within the mouse cortex were examined using TEM following a standardized protocol. Briefly, a 1 mm^3^ sample of mouse cortex tissue was fixed for 12 h in PBS containing 2.5% glutaraldehyde and 2% paraformaldehyde. Samples were then incubated for 1 h in PBS containing 1% osmium tetroxide. Following embedding in epoxy resin, 60 nm ultrathin sections were obtained using an ultramicrotome (UC7, Leica). Sections were then observed and photographed using a TEM (HT7700, Hitachi, Japan). ImageJ software was used to analyze at least five nonoverlapping fields of view per section.

### 2.15 ELISA

Inflammatory cytokine levels were assessed using commercial ELISA kits (Elabscience Biotechnology Co. Ltd.) following the manufacturer's protocols.

### 2.16 Cell Culture and Administration

BV2 and HT22 cells (Pricella Life Science & Technology) were maintained in a high‐glucose medium supplemented with 100 U/mL penicillin, 100 μg/mL streptomycin, and 10% fetal bovine serum. To elucidate the potential mechanisms by which OX‐A promotes microglial‐mediated neuronal death in vitro, we established a Transwell coculture system using BV2 microglial cells and HT22 neurons. BV2 cells were initially treated with OX‐A (1 μM; MedChemExpress, USA) and the NF‐κB agonist PapRIV (5 μM; MedChemExpress) for 24 h, followed by LPS (1 μg/mL) exposure for 24 h. Prior to coculture, BV2 cells were washed three times with PBS to remove residual LPS and prevent it from directly affecting HT22 cells. The coculture system was assembled with 2 × 10^5^ BV2 cells in the upper Transwell chamber and 5 × 10^5^ HT22 cells in the lower chamber. Following 24 h of coculture, the neuroprotective efficacy of OX‐A was assessed through multiple analytical methods, including CCK‐8, ELISA, and Western blot analysis.

### 2.17 Transwell Migration Assay

Transwell assays were conducted to evaluate the migratory capacity of HT22 cells. Following coculture with BV2 cells, HT22 cells were resuspended and seeded in the upper chamber. Following 24 h of incubation, the chambers were fixed with 4% paraformaldehyde for 1 h. The cells in the inserts were then carefully removed, and the inserts were washed and allowed to dry. Migrated cells were observed and recorded using an inverted microscope.

### 2.18 MMP Assessment

In accordance with the manufacturer's guidelines, the alterations in MMP were quantified utilizing the JC‐1 assay kit (Solarbio). HT22 cells in the coculture setup were treated with the JC‐1 dye for 30 min. Subsequently, the cells underwent three washes with PBS, and images were then captured using a fluorescence microscope.

### 2.19 Flow Cytometry (FCM) Analysis

HT22 cells were harvested after coculture and resuspended in PBS. To assess apoptosis, cells were incubated with 5 μL of Annexin V for 10 min, followed by 5 μL of 7‐AAD for 5 min in the dark. Subsequently, samples were analyzed via flow cytometry. Fluorescence‐minus‐one (FMO) controls were implemented to accurately establish gating parameters for flow cytometric data acquisition. For analysis of BV2 microglial polarization, cells were labeled with phycoerythrin‐conjugated anti‐CD206 or anti‐CD86 antibodies, concurrent with staining using FITC‐conjugated anti‐IBA1 antibody (BioLegend, USA) to identify microglia. This antibody cocktail was incubated with the cells at 4°C for 30 min. Following incubation, cells were washed three times with 2% bovine serum albumin before flow cytometric analysis.

### 2.20 Cell Viability Analysis

In accordance with the manufacturer's guidelines, the Cell Counting Kit‐8 (CCK8, Beyotime) was employed to assess cell viability. Following treatment, HT22 cells were seeded in a 96‐well plate and cocultured with BV2 cells for 24 h, followed by the addition of 10 μL of CCK‐8 for 45 min. After washing three times with PBS, cell viability was measured using a microplate spectrophotometer.

### 2.21 Western Blot Analysis

Total protein was extracted from tissue or cells using a commercial RIPA kit. Subsequently, equal amounts of protein (30 μg per group) were separated and then transferred onto PVDF membranes. Following transfer, the membranes were blocked with 5% milk for 2 h and then exposed to primary antibodies targeting OX1R (183701AP, Proteintech), MAP2 (ab254264, Abcam, USA), OX‐A (ab6214, Abcam), GAP43 (ab277627, Abcam), Bcl‐xL (ab270253, Abcam), NeuN (ab104224, Abcam), SYP (ab32127, Abcam), PSD95 (ab238135, Abcam), p‐p65 (ab76302, Abcam), p‐IκBα (ab92700, Abcam), iNOS (ab178945, Abcam), and Arg‐1 (#93668, Cell Signaling Technology). Subsequently, membranes were exposed to HRP‐conjugated secondary antibodies for 2 h. Bands were quantified with Image J software.

### 2.22 Quantitative RT‐PCR (qRT‐PCR)

Total RNA was extracted from tissues or cells using a commercial RNA extraction kit (TransGen Bio, China). The RNA was then reverse‐transcribed into cDNA. Subsequently, quantitative PCR was performed using PCR Master Mix. The relative expression levels of mRNA were normalized to GAPDH as an internal control and calculated using the 2^−ΔΔCT^ method. The primer sequences were as follows: TNF‐α: Forward: 5′‐CTCATGCACCACCATCAAGG‐3′, Reverse: 5′‐ACCTGACCACTCTCCCTTTG‐3′; IL‐10: Forward: 5′‐CAGAGCCACATGCTCCTAGA‐3′, Reverse: 5′‐GTCCAGCTGGTCCTTTGTTT‐3′; IL‐1β: Forward: 5′‐CCCTTGACTTGGGCTGT‐3′, Reverse: 5′‐CGAGATGCTGCTGTGAGA‐3′; and GAPDH: Forward: 5′‐AACTTTGGCATTGTGGAAGG‐3′, Reverse: 5′‐GG ATGCAGGGATGATGTTCT‐3′.

### 2.23 Statistical Analysis

The Shapiro–Wilk test was used to assess data normality. Results for normally distributed data were presented as mean ± standard deviation. One‐way ANOVA with Duncan's post hoc test was employed for between‐group comparisons, and two‐way repeated measures ANOVA with Tukey's post hoc test analyzed behavioral data. The Kruskal–Wallis test was used to assess group differences for data not following a normal distribution. Statistical analyses were performed using SPSS software. A threshold of *p* < 0.05 was established to determine statistical significance.

## 3 Results

### 3.1 tPCS Attenuates Neuroinflammation, Cerebral Damage, and Neurological Deficits Following TBI

**FIGURE 1:**
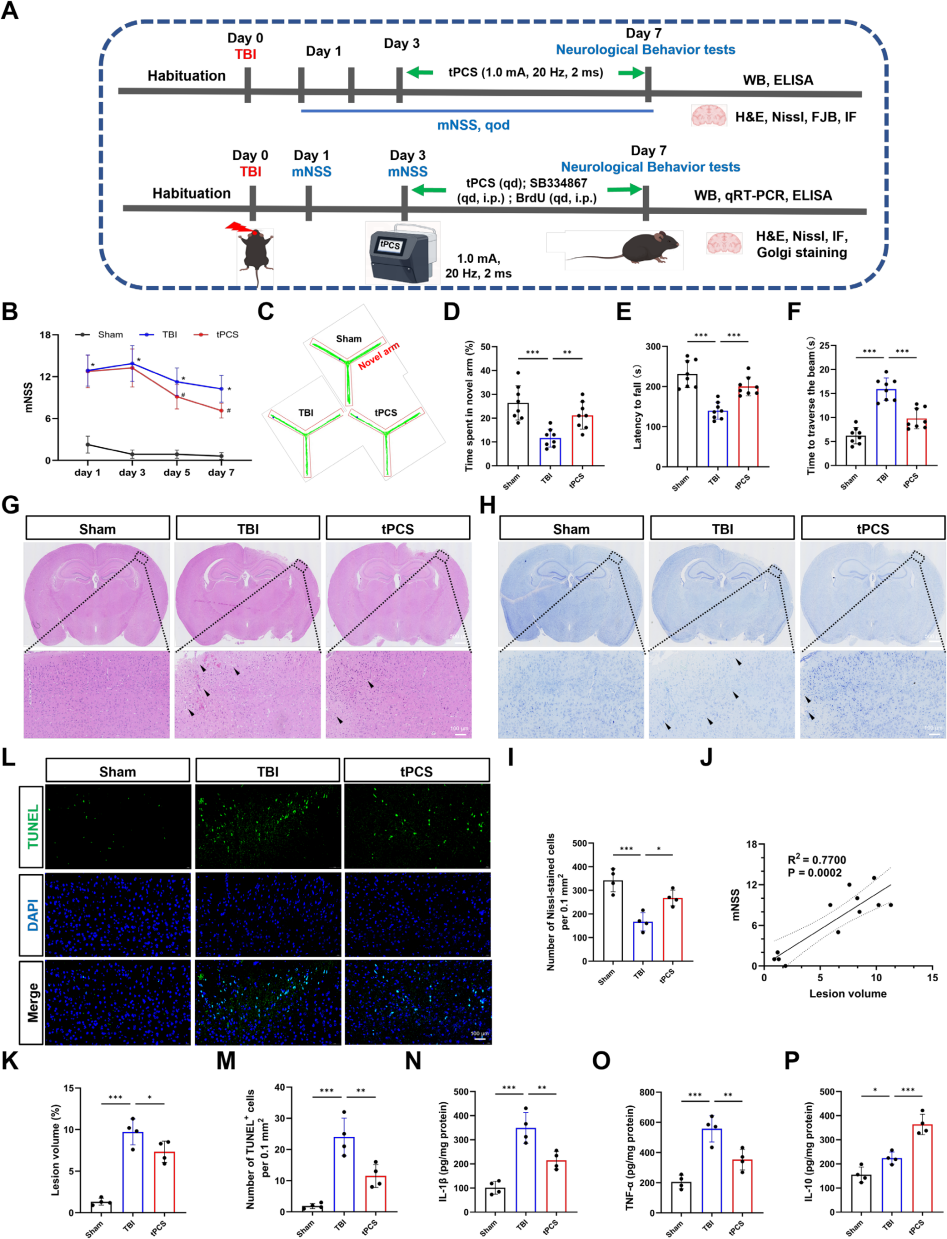
Protective effects of tPCS on neurological function, pathological alterations, and inflammatory response following TBI. (A) Experimental procedure timeline. (B) Neurological function in mice was assessed using the mNSS at 1, 3, 5, and 7 dpo (*n* = 8). (C) Representative exploration routes in the Y maze. (D) Time spent in the novel arm of the Y‐maze (*n* = 8). (E) Beam‐balance test measuring the time required to traverse the beam (*n* = 8). (F) Rotarod test measuring the latency to fall (*n* = 8). (G) Representative scanning images and photomicrographs of H&E‐stained sections at 7 dpo (*n* = 4). Arrows point to the neuronal edema, vacuolation, and degeneration. (H) Representative scanning images and photomicrographs of Nissl‐stained sections at 7 dpo (*n* = 4). Arrows highlight neuronal vacuolation and degeneration. (G) Quantification of Nissl‐stained cells. (I–K) Quantitative analysis of Nissl‐stained cells, lesion volume, and correlation analysis between lesion volume and mNSS. (L) Representative images of TUNEL staining at 7 dpo. (M) Quantification of TUNEL‐stained cells (*n* = 4). (N–P) The levels of inflammatory mediators including IL‐1β, TNF‐α, and IL‐10 in the peri‐lesional cortex were detected by ELISA (*n* = 4). Scale bar = 100 μm. All values are mean ± SD. *p < 0.05, **p < 0.01, ***p < 0.001.

Figure 1A illustrates the experimental timeline. To evaluate the impact of tPCS on neurological function post‐TBI, we employed the mNSS. At 1 dpo, before tPCS intervention, both TBI and tPCS‐designated TBI groups demonstrated higher mNSS compared to the Sham group (Figure 1B, *p* < 0.001). At 5 and 7 dpo, the untreated TBI group continued to exhibit elevated scores relative to the Sham group (Figure 1B, *p* < 0.001), indicating persistent neurological deficits. Following TBI in mice, a notable reduction in mNSS was observed after tPCS treatment (*p* < 0.001), suggesting that tPCS may facilitate neurological recovery post‐TBI. Given that motor and cognitive impairments represent prominent clinical features in TBI patients [44], we subsequently investigated the effects of tPCS on these deficits. At 7 dpo, behavioral assessments revealed significant deficits in the TBI group compared to Sham controls, as evidenced by reduced C‐arm exploration time in the Y‐maze, increased traversal time in the beam‐balance test, and decreased latency to fall in the rotarod test (*p* < 0.001; Figure 1C–F). Notably, mice receiving a 5‐day tPCS treatment protocol exhibited marked improvements across these parameters compared to untreated TBI mice, demonstrating increased exploration time in the novel arm, reduced traversal time, and prolonged latency to fall (*p* < 0.001). These findings indicate that tPCS effectively ameliorates motor and working memory deficits following TBI.

To ascertain the impact of tPCS on the histopathological changes in the peri‐lesional cortex of TBI mice, we conducted comprehensive analyses using HE, Nissl, and TUNEL staining. Examination of the Sham group revealed well‐organized cerebral tissue architecture with normal neuronal arrangements. In contrast, the TBI group exhibited severe structural disruptions, characterized by disorganized and fragmented tissue, extensive vacuolization, and atrophy (Figure 1G–M). Quantitative analysis revealed a significant reduction in Nissl‐positive cells and a substantial rise in TUNEL‐positive cells in TBI cohorts in comparison to the Sham group. The TBI group also showed significantly increased lesion volume (*p* < 0.001), which demonstrated a moderate positive correlation with mNSS (*R* ^2^ = 0.7700, *p* < 0.001). Notably, tPCS intervention significantly mitigated these post‐TBI histopathological alterations, as evidenced by increased Nissl‐positive cell counts (*p* < 0.05), decreased TUNEL‐positive cell numbers (*p* < 0.01), and reduced lesion volume (*p* < 0.01).

Given that neuroinflammation is a key driver of neuronal damage and apoptosis following TBI, and considering previous studies indicating tPCS's anti‐inflammatory effects in stroke, we investigated tPCS's impact on post‐TBI inflammatory response by measuring cytokine levels via ELISA. Our analysis revealed that TBI induced significant elevations in both pro‐inflammatory cytokines, including IL‐1β and TNF‐α (Figure 1N–P, *p* < 0.001), and the anti‐inflammatory cytokine IL‐10 in the perilesional cortex (Figure 1N, *p* < 0.05). Notably, administration of tPCS demonstrated dual regulatory effects on the inflammatory response: it effectively decreased pro‐inflammatory cytokine levels (*p* < 0.001) while simultaneously augmenting IL‐10 expression (*p* < 0.01). These findings collectively suggest that tPCS may promote neurological recovery and attenuate tissue damage by modulating neuroinflammatory signaling.

### 3.2 tPCS Enhanced the Expression of OX‐A, OX1R, MAP2, GAP43, and TGF‐β Following TBI

**FIGURE 2:**
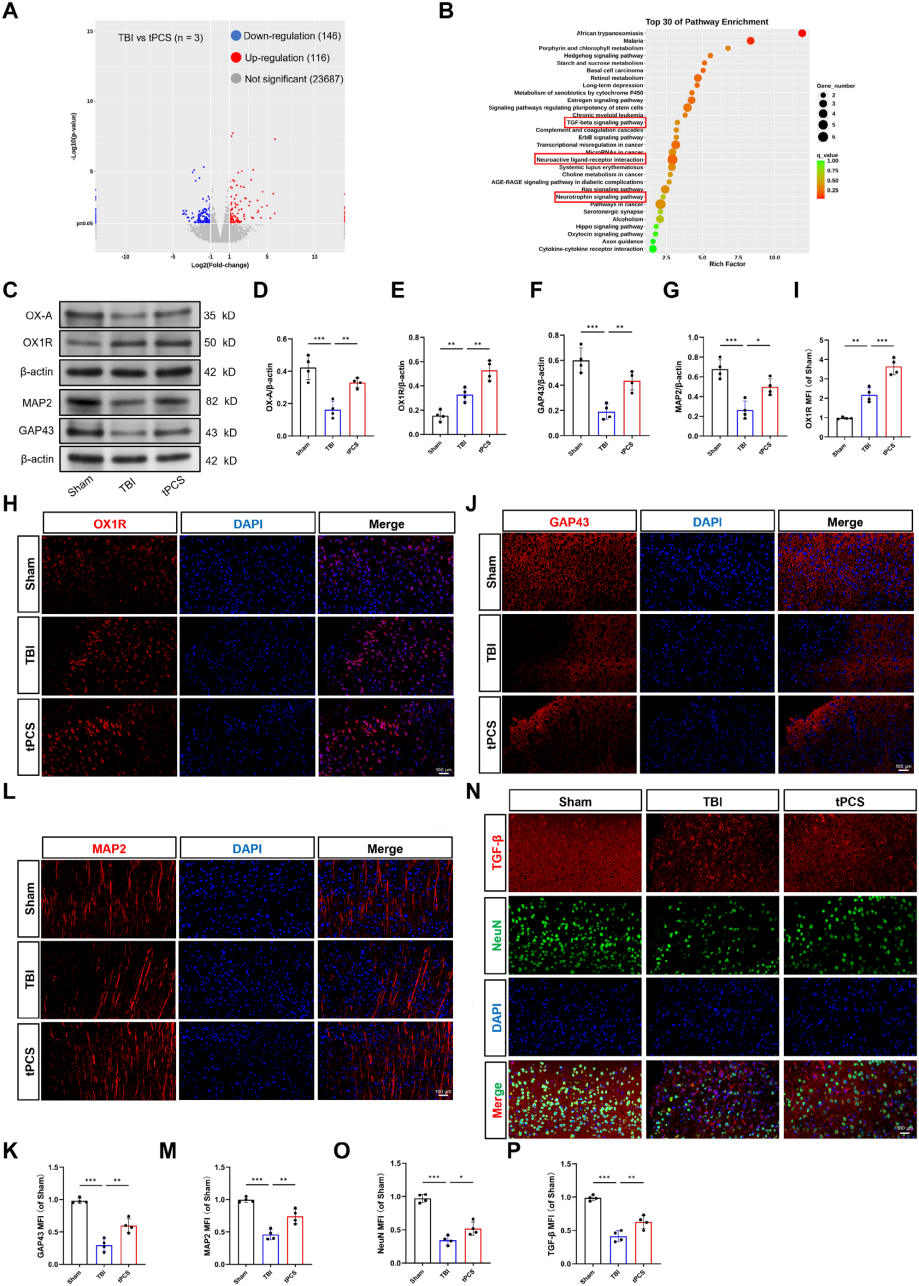
The impact of tPCS on the expression of OX‐A, OX1R, MAP2, GAP43, and TGF‐β in the peri‐lesional cortex of TBI mice. (A, B) Transcriptome sequencing identified DEGs between the TBI and tPCS groups, as visualized in a volcano plot, and KEGG enrichment analysis was performed on these DEGs (*n* = 3). (C–G) Representative Western blot images and quantification illustrate the levels of OX‐A, OX1R, GAP43, and MAP2 in the peri‐lesional cortex at 7 dpo (*n* = 4), with β‐actin serving as the loading control. The original blots are presented in the Appendix S1. (H–M) Representative IF images and quantification depict the expression levels of OX1R, GAP43, and MAP2 at 7 dpo (*n* = 4). (N–P) Representative IF images and quantitative analysis of NeuN and TGF‐β expression levels in the peri‐lesional cortex (*n* = 4). Scale bar =100 μm. All values are mean ± SD. *p < 0.05, **p < 0.01, ***p < 0.001.

To investigate the neuroprotective mechanisms of tPCS, we performed transcriptomic sequencing on peri‐lesional cortex tissue from TBI and tPCS‐treated mice. Analysis of 23,949 readings identified 262 DEGs, comprising 146 upregulated and 116 downregulated genes (Figure 2A). KEGG enrichment analysis of these DEGs revealed significant enrichment in several signaling pathways, including neuroactive ligand‐receptor interaction, long‐term depression, neurotrophin signaling pathway, and TGF‐β signaling pathway (Figure 2B).

Previous research has established the crucial regulatory role of OX‐A in anti‐inflammation and neurorepair [45], and our preliminary investigations corroborate its significance in recovery following TBI [30]. Given the importance of the neurotrophin factor and TGF‐β signaling pathways in neural repair and neurogenesis, we investigated whether the neuroprotective effects of tPCS are associated with OX‐A‐enhanced expression of neurorepair‐related markers following TBI. To this end, we assessed the expression levels of OX‐A, OX1R, MAP2, and GAP43, using Western blot analysis. Our results revealed that, compared to the Sham group, the TBI group exhibited significantly decreased levels of OX‐A, MAP2, and GAP43 (Figure 2C–G, *p* < 0.001), and increased levels of OX1R (*p* < 0.01). OX2R levels, however, remained unchanged (Figure S1, *p* > 0.05). Notably, tPCS intervention led to a significant increase in the expression of all these proteins (*p* < 0.01). These findings were further validated by IF staining, confirming the enhanced expression of OX1R and the reduction in MAP2 and GAP43 expression following TBI. Furthermore, tPCS treatment promoted the expression of these proteins (Figure 2H–M, *p* < 0.01). Subsequently, we examined the expression of TGF‐β, a key regulator involved in neurorepair [46]. Our analysis demonstrated that TBI significantly reduced the expression of both NeuN and TGF‐β (Figure 2N–P, *p* < 0.001), an effect that was reversed by tPCS. Taken together, these results suggest that the neuroprotective effects of tPCS may be mediated, at least in part, through the enhancement of OX‐A and neurogrowth‐associated protein expression in the peri‐lesional cortex.

### 3.3 tPCS Mitigated Neuronal Apoptosis and Neurological Deficits via OX‐A/OX1R in TBI Mice

**FIGURE 3:**
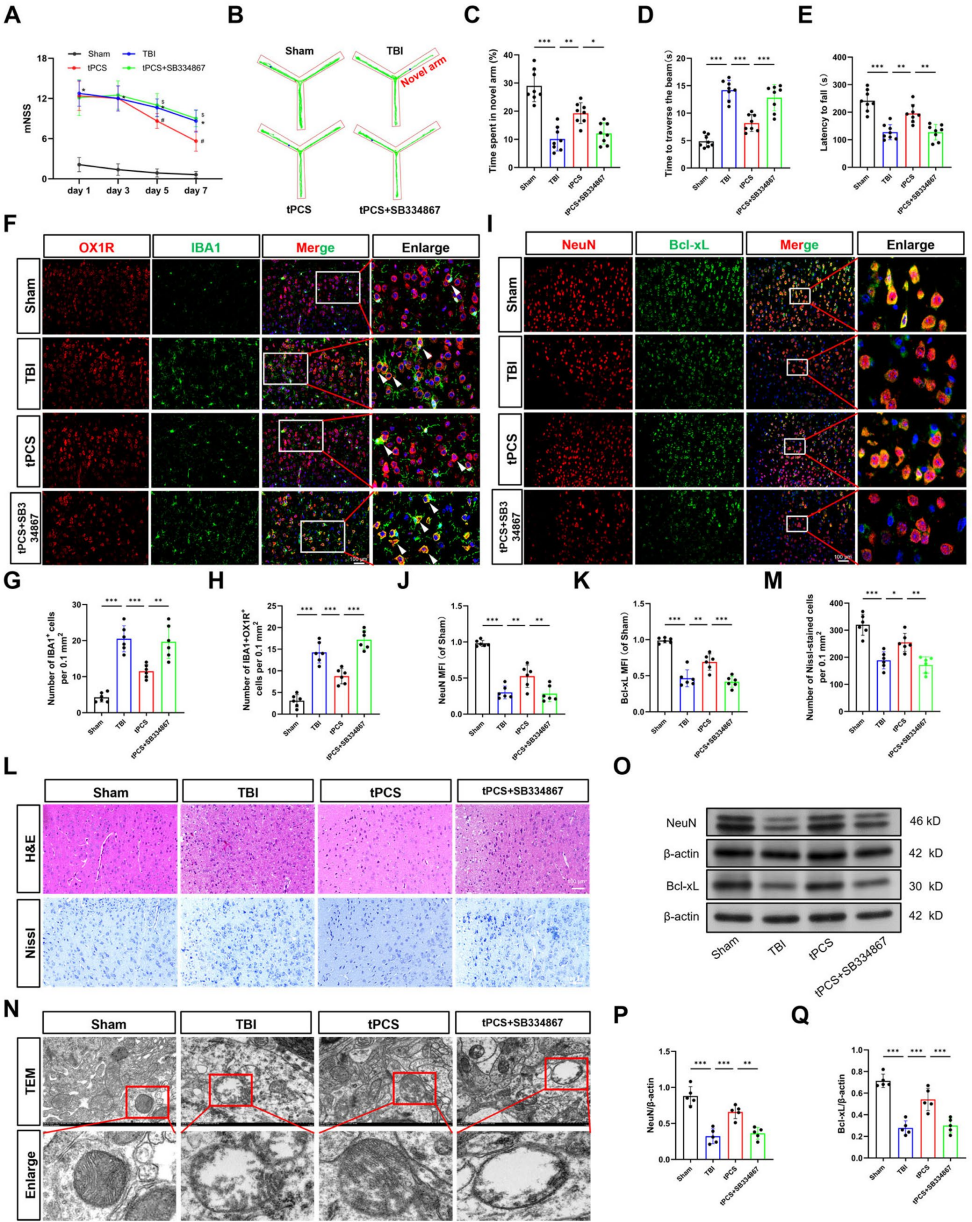
tPCS attenuated neuronal apoptosis and ameliorated neurological deficits via OX‐A/OX1R in TBI mice. (A–E) Motor function of TBI mice was assessed using Y‐maze, beam‐balance test, and rotarod test (*n* = 8). (F–H) Representative IF images and quantitative analysis of IBA1 and OX1R expression levels in the peri‐lesional cortex (*n* = 6). The arrows represent OX1R+IBA1‐positive cells. (I–K) Representative IF images and quantitative analysis of Bcl‐xL and NeuN (*n* = 6). (L) Representative images of the cortical region were obtained using HE staining, Nissl staining, and transmission electron microscopy. (M) Quantification of Nissl‐stained cells (*n* = 6). (N) Representative images of the cortical region were obtained using transmission electron microscopy. (O–Q) Representative Western blot bands and quantitative analysis demonstrating Bcl‐xL and NeuN levels in the peri‐lesional cortex (*n* = 5), with β‐actin employed as the loading control. Scale bar = 100 μm. The original blots are presented in Appendix S1. All values are mean ± SD. *p < 0.05, **p < 0.01, ***p < 0.001.

To investigate whether tPCS‐induced neurological functional recovery following TBI is mediated by OX‐A/OX1R signaling, we administered the OX1R antagonist SB334867 to tPCS‐treated mice and evaluated their neurobehavioral outcomes. Consistent with our initial findings, tPCS‐treated mice exhibited significant improvements in motor and cognitive functions at 7 dpo, as evidenced by reduced mNSS scores, increased time spent in the novel arm, decreased traversal time, and extended fall latency (Figure 3A–E, *p* < 0.001). Furthermore, results from the MWM and NOR experiments indicated that tPCS mitigated TBI‐induced learning and memory impairments, as evidenced by a decreased escape latency and increased time spent in the target quadrant in the MWM, and an increased recognition index in the NOR (Figure S2A–F). Notably, these beneficial effects were substantially reversed following SB334867 administration.

Considering the established role of microglial hyperactivation in post‐TBI neuroinflammation, we investigated whether tPCS‐mediated OX‐A upregulation affected microglial activation. Dual IF staining for the microglial marker IBA1 and OX1R revealed pronounced microglial activation in the peri‐lesional cortex of TBI mice (*p* < 0.001), as evidenced by hypertrophic soma and retracted processes (Figure 3F). This activation was further characterized by a notable increase in IBA1‐positive cells and IBA1+/OX1R+ double‐positive cells (Figure 3G,H, *p* < 0.001). Importantly, tPCS therapy effectively reduced the number of activated microglia (*p* < 0.01) as well as IBA1+/OX1R+ cells (*p* < 0.001). However, SB334867 administration negated these tPCS‐induced anti‐inflammatory effects.

To assess the impact of tPCS on neurodegeneration and apoptosis following TBI, we performed histopathological staining, TEM, and dual IF staining for NeuN and Bcl‐xL in the peri‐lesional cortex. Dual IF staining revealed a significant decrease in the expression levels of both NeuN and Bcl‐xL following TBI (Figure 3I–K, *p* < 0.001), while tPCS upregulated the expression of NeuN (*p* < 0.01) and Bcl‐xL (*p* < 0.001). Consistent with these observations, histopathological analysis revealed that tPCS effectively attenuated neurodegeneration and neuronal loss (Figure 3L,M). Further corroborating this finding, TEM confirmed the neuroprotective effect of tPCS on mitochondrial damage, as evidenced by diminished mitochondrial swelling and cristae loss (Figure 3N). Similarly, tPCS also effectively alleviated neurodegenerative changes and neuronal apoptosis in the hippocampus (Figure S2G–Q). To further validate these findings, we performed Western blot analysis, which corroborated the IF staining results, demonstrating that tPCS enhanced the expression of NeuN and Bcl‐xL (Figure 3O–Q, *p* < 0.001). Similar trends were observed in the hippocampus as in the peri‐lesional cortex (Figure S2R–V, *p* < 0.001). Importantly, SB334867 abolished these neuroprotective effects and significantly attenuated NeuN and Bcl‐xL expression (*p* < 0.01). Taken together, these findings suggest that tPCS alleviates neurological deficits after TBI by reducing microglial activation and neuronal apoptosis via the OX‐A/OX1R pathway.

### 3.4 tPCS Enhances the Expression of Neurorepair‐Associated Markers and Neuroplasticity Following TBI via the OX‐A/OX1R Pathway

**FIGURE 4:**
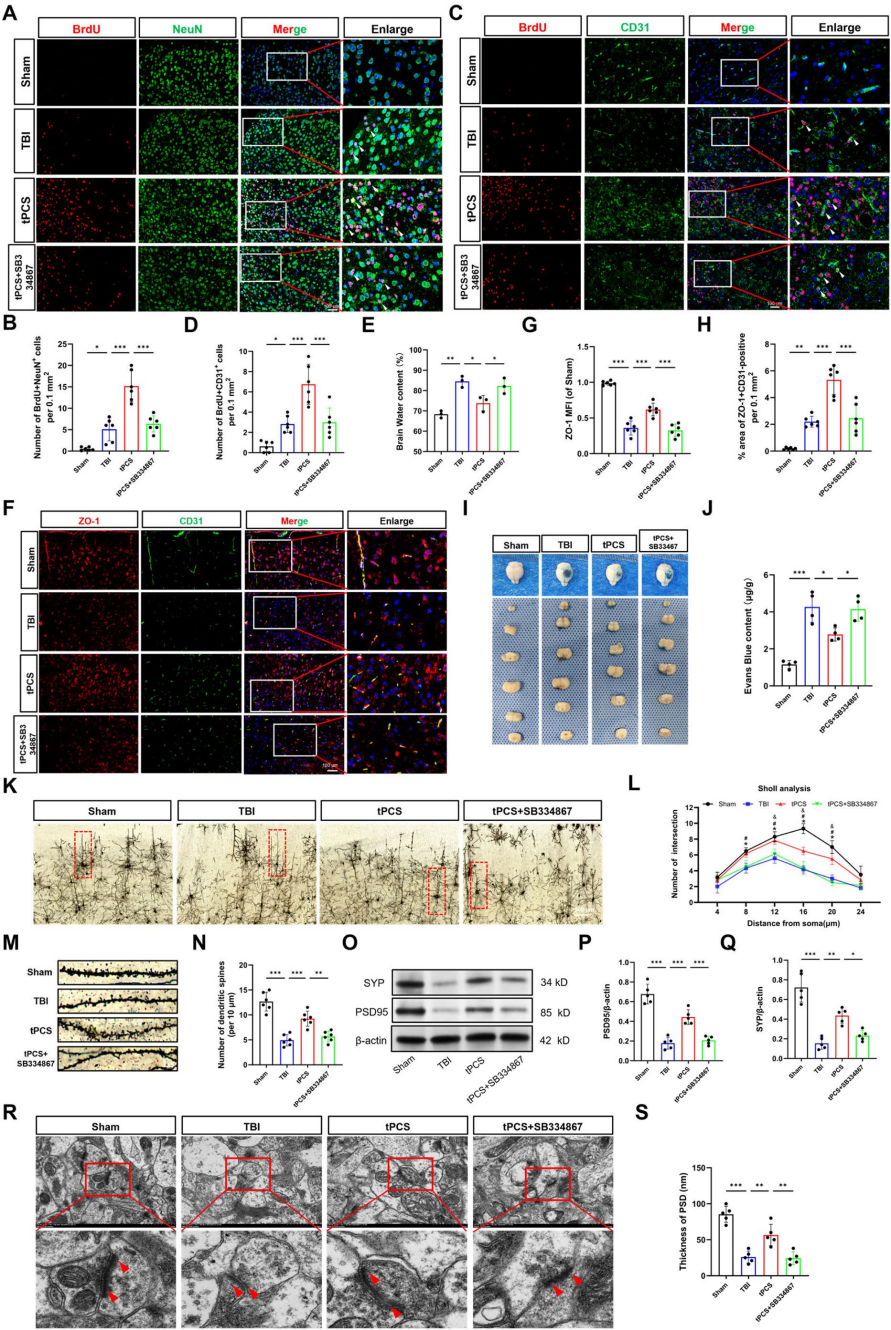
tPCS enhances the expression of neurorepair‐related markers and neuroplasticity following TBI via the OX‐A/OX1R pathway. (A, B) Representative IF images and quantification of NeuN+BrdU‐positive cells in the peri‐lesional cortex (*n* = 6). The arrows represent NeuN+BrdU‐positive cells. Scale bar = 100 μm. (C, D) Representative IF images and quantification of CD31+BrdU‐positive cells (*n* = 6). The arrows represent CD31+BrdU positive cells. Scale bar = 100 μm. (E) Quantification of the brain water content (*n* = 3). (F–H) Representative IF images and quantitative analysis of ZO‐1 and CD31 in the peri‐lesional cortex (*n* = 6). Scale bar = 100 μm. (I, J) Representative images and quantitative analysis of Evans Blue staining at 7dpo (*n* = 4). (K, L) Representative images of Golgi staining and Sholl analysis in the peri‐lesional cortex (*n* = 6). The red dotted box represents the neurons selected for Sholl analysis. Scale bar =50 μm. (M, N) Representative imaging of dendrites and quantification of dendritic density (*n* = 6). (O–Q) SYP and PSD95 proteins were measured and quantified at 7 dpo by Western blot (*n* = 5), with β‐actin serving as a loading control. The original blots are presented in Appendix S1. (R, S) Representative TEM images and quantification of postsynaptic densities in the perilesional cortex. Scale bar = 1 μm. All values are mean ± SD. *p < 0.05, **p < 0.01, ***p < 0.001.

Extant literature emphasizes that microglia are not only pivotal in modulating the immune‐inflammatory response post‐TBI but also play an instrumental role in neurorepair and the reconstitution of neurological function [47]. To investigate the impact of tPCS on the neurovascular unit, we first conducted BrdU+NeuN and BrdU+CD31 dual IF staining. Within the TBI lesion area, NeuN and CD31 were utilized to identify neurons and endothelial cells, respectively, while BrdU staining identified proliferating cells, with colocalization indicating newly formed neurons and blood vessels. The TBI group exhibited elevated numbers of BrdU+/NeuN+ and BrdU+/CD31+ cells in the peri‐lesional cortex compared to the Sham group (*p* < 0.05), as shown in Figure 4A–D. Notably, tPCS treatment further enhanced these cell populations significantly (*p* < 0.001). Analysis of contralateral ROIs as an internal control yielded a pattern consistent with those observed in the peri‐lesional cortex (Figure S3A–F), and hippocampal data also mirrored these perilesional cortical changes (Figure S2W–Z). However, the administration of SB334867 attenuated these increases, suggesting that tPCS‐induced upregulation of neurorepair‐associated markers is potentially mediated by the OX‐A/OX1R pathway. Additional evaluation of astrocyte and microglial cell proliferation in the peri‐lesional cortex through BrdU+GFAP and BrdU+IBA1 dual IF staining revealed no significant effects of tPCS (Figure S4A–F).

To evaluate the protective effects of tPCS on the BBB, we employed EB staining, brain water content measurement, and ZO‐1/CD31 dual IF staining, considering the critical role of the neurovascular unit in BBB integrity. In the TBI group, there were significant increases in brain water content (*p* < 0.01) and EB extravasation (*p* < 0.001), accompanied by decreased expression of ZO‐1 and a reduced vascular coverage area (Figure 4E–J, *p* < 0.001), indicating TBI‐induced BBB hyperpermeability. Treatment with tPCS significantly ameliorated these alterations, as evidenced by reduced EB extravasation and brain water content (*p* < 0.05), alongside enhanced ZO‐1 expression and increased vascular coverage area (*p* < 0.001), suggesting improved BBB integrity. However, the administration of SB334867 reversed these beneficial effects.

Subsequent investigations into neuronal morphological and synaptic plasticity in the peri‐lesional cortex revealed significant alterations following TBI. Specifically, Sholl analysis demonstrated a marked reduction in both dendritic intersections and dendritic spine density in the TBI group (Figure 4K–N, *p* < 0.001), indicative of impaired neuronal complexity and synaptic plasticity. Notably, tPCS significantly ameliorated these deficits, suggesting an enhancement of neuronal morphology and synaptic plasticity recovery post‐TBI. However, this tPCS‐induced improvement was substantially attenuated by SB334867 administration. Consistent with the Golgi staining results, Western blot analysis further revealed that tPCS treatment led to increased expression levels of SYP and PSD95 (Figure 4O–Q, *p* < 0.01). Furthermore, TEM analysis indicated that tPCS significantly increased PSD thickness (Figure 4R,S, *p* < 0.01), an effect that was abolished by SB334867. Collectively, these findings suggest that tPCS enhances the expression of neurorepair‐associated markers and neuroplasticity following TBI, potentially through activation of the OX‐A/OX1R pathway.

### 3.5 tPCS Modulates M1/M2 Microglia Polarization via OX‐A/OX1R/NF‐κB Pathway After TBI

**FIGURE 5:**
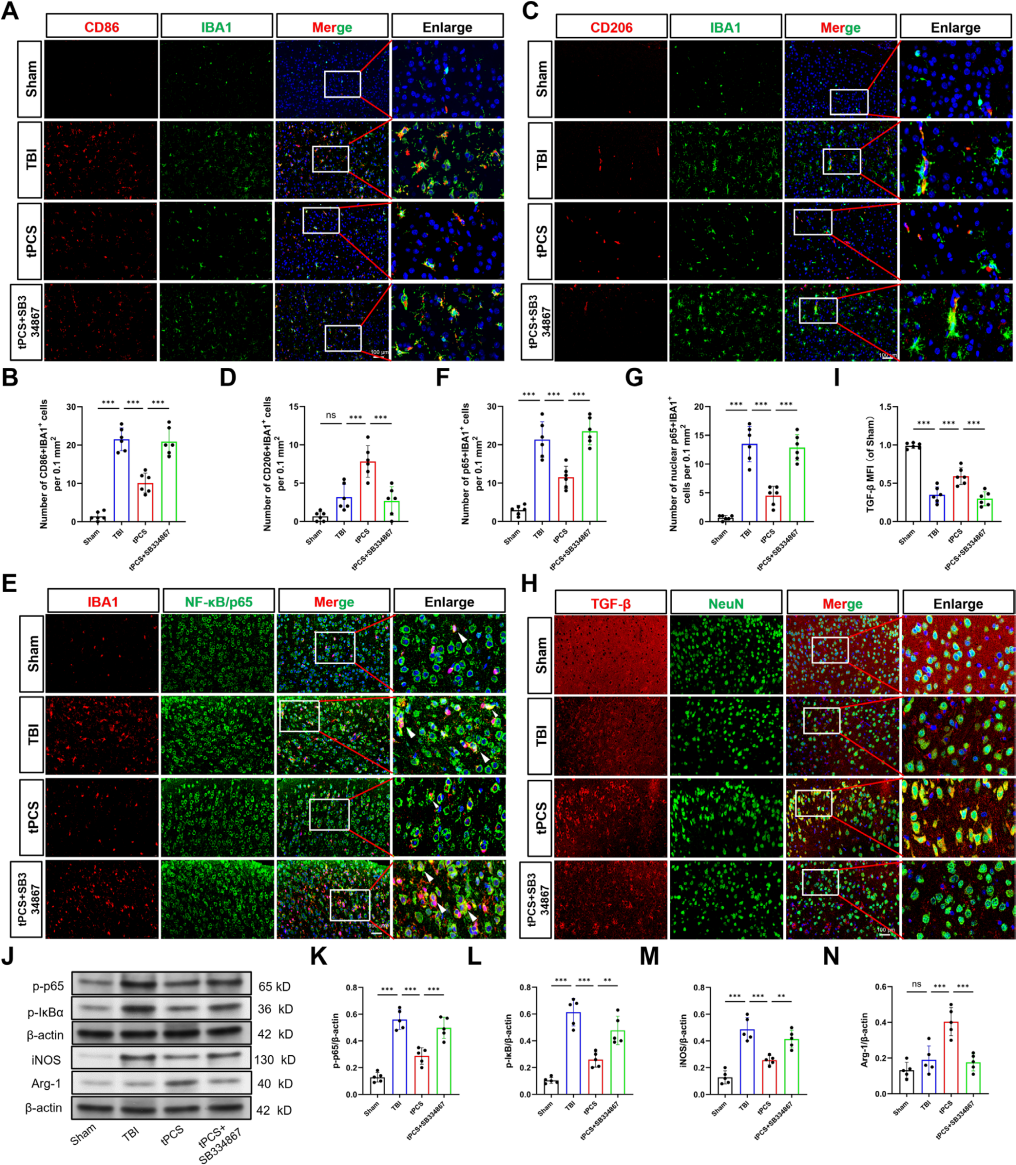
Treatment with tPCS modulates M1/M2 microglia polarization via OX‐A/OX1R‐mediated NF‐κB pathway following TBI. (A, B) Representative IF images and quantification of CD86+IBA1‐positive cells in the peri‐lesional cortex (*n* = 6). (C, D) Representative IF image and quantification of CD206+IBA1‐positive cells (*n* = 6). (E–G) Representative immunofluorescence image and quantification of p65+IBA1‐positive cells (*n* = 6). (H, I) Representative IF image and quantitative analysis of NeuN and TGF‐β (*n* = 6). (J–N) Representative Western blot bands and quantification showing levels of phosphorylated (p)‐p65, p‐IκBα, iNOS, and Arg‐1 proteins in the peri‐lesional cortex (*n* = 5), with β‐actin serving as a loading control. All values are mean ± SD. Scale bar = 100 μm. The original blots are presented in Appendix S1. *p < 0.05, **p < 0.01, ***p < 0.001.

Microglial polarization, specifically the modulation of M1/M2 phenotypes, is recognized as a critical factor in mitigating neuroinflammation and improving functional outcomes following TBI [8]. To investigate the impact of tPCS on microglial polarization, we conducted dual IF staining to quantify IBA1+CD86+ (M1 phenotype) and IBA1+CD206+ (M2 phenotype) cells. Analysis at 7 dpo revealed a significant increase in M1 microglia (IBA1+/CD86+) in the peri‐lesional cortex of the TBI group (*p* < 0.001), while the number of M2 microglia (IBA1+/CD206+) remained statistically unchanged (*p* > 0.05) (Figure 5A–D). Notably, tPCS administration resulted in a significant reduction in M1 microglia (*p* < 0.01) and a concurrent increase in M2 microglia (*p* < 0.001). Similar trends were observed in the contralateral ROIs, which served as an internal control (Figure S5A–F). Furthermore, treatment with SB334867 abolished these tPCS‐induced effects, suggesting that tPCS promotes the transition of microglia from the M1 to M2 phenotype via activating the OX‐A/OX1R pathway.

The NF‐κB inflammatory pathway is a key regulator of microglial polarization and neuroinflammation [48]. Activation of this pathway is characterized by nuclear translocation of p65. To elucidate whether tPCS influences microglial phenotypes post‐TBI through this mechanism, we assessed p65 expression in microglia and the expression of NF‐κB pathway‐related proteins in the peri‐lesional cortex. Our dual IF results demonstrated a significant increase in nuclear translocation of microglial p65 (*p* < 0.001) and a concomitant reduction in TGF‐β secretion (*p* < 0.001) in TBI mice (*p* < 0.001, Figure 5E–I). Complementary Western blot analysis further confirmed NF‐κB pathway activation following TBI, as evidenced by elevated levels of p‐p65, p‐IκBα, and iNOS (*p* < 0.001), while Arg‐1 expression remained unchanged (*p* > 0.05, Figure 5J–N). In contrast, tPCS therapy attenuated p65 nuclear translocation and levels of these inflammatory markers. Simultaneously, tPCS enhanced the expression of M2 microglial marker Arg‐1 and TGF‐β (*p* < 0.001), suggesting inhibition of NF‐κB activation and a promotion of M2 microglial polarization. SB334867 treatment reversed these effects, confirming that tPCS promotes the M1 to M2 microglial shift after TBI via the OX‐A/OX1R/NF‐κB pathway.

### 3.6 OX‐A Attenuated the Release of Pro‐Inflammatory Mediators in LPS‐Activated Microglia

**FIGURE 6:**
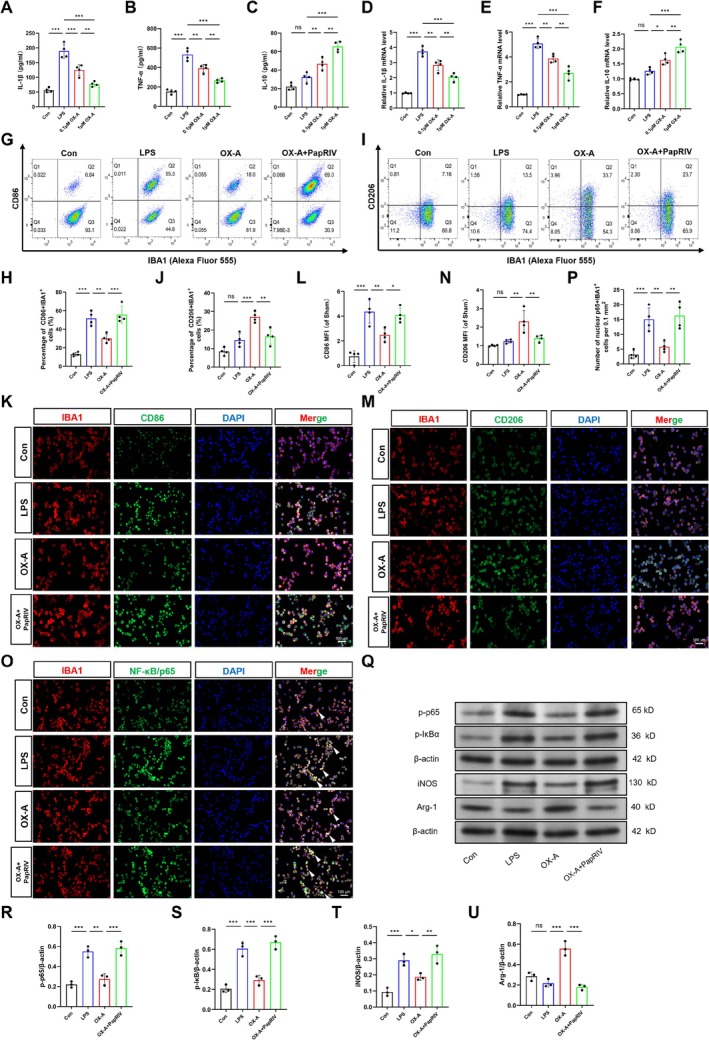
OX‐A attenuated the production of pro‐inflammatory mediators in LPS‐activated microglia. (A–C) The level of inflammatory mediators, including IL‐1β, TNF‐α, and IL‐10, was quantified using ELISA (*n* = 4 independent measures). (D–F) The mRNA expression level of IL‐1β, TNF‐α, and IL‐10 was measured via qRT‐PCR (*n* = 4 in each group). (G, H) Representative FCM images and quantification depicting IBA1+CD68‐positive microglia (*n* = 4 independent measures). (I, J) Representative FCM images and quantification depicting IBA1+CD206‐positive microglia (*n* = 4 independent measures). (K, L) Representative IF images and quantification depicting IBA1+CD68‐positive microglia (*n* = 4 independent measures). (M, N) Representative IF images and quantification depicting IBA1+CD206‐positive microglia (*n* = 4 independent measures). (O, P) Representative IF images and quantification depicting IBA1+p65‐positive microglia (*n* = 4 independent measures). (Q–U) Representative Western blot bands and quantification showing p‐p65, p‐IκBα, iNOS, and Arg‐1 protein levels (*n* = 3 independent measures). β‐actin was utilized as a loading control. All values are mean ± SD. The original blots are presented in Appendix S1. *p < 0.05, **p < 0.01, ***p < 0.001.

To investigate the effects of OX‐A on cytokine expression in LPS‐activated microglial cells, we employed an in vitro neuroinflammation model by stimulating BV2 cells with LPS. Initial ELISA analysis revealed that LPS treatment significantly increased the levels of the pro‐inflammatory cytokines IL‐1β and TNF‐α compared to the control group (*p* < 0.001). In contrast, the expression of the anti‐inflammatory cytokine IL‐10 remained unchanged (*p* > 0.05) (Figure 6A–C). Notably, pretreatment with OX‐A induced a dose‐dependent reduction in the levels of these pro‐inflammatory cytokines relative to the LPS‐treated group, while concurrently increasing IL‐10 levels (*p* < 0.01). These observations were further substantiated by qRT‐PCR analysis, which demonstrated that OX‐A pretreatment dose‐dependently suppressed LPS‐stimulated upregulation of IL‐1β and TNF‐α mRNA, and enhanced IL‐10 mRNA expression (Figure 6D–F, *p* < 0.01).

To further investigate these findings, we pretreated microglia with the NF‐κB activator PapRIV (5 μM) [49] and OX‐A (1 μM) in vitro, followed by FCM, IF, and Western blot analyses. Consistent with expectations, LPS exposure significantly increased the expression of the M1 surface marker CD86 (*p* < 0.001) while maintaining stable levels of the M2 surface marker CD206 (*p* > 0.05, Figure 6G–J). These findings were corroborated by Dual IF staining of IBA1+CD86 and IBA1+CD206 (Figure 6K–N), confirming M1 polarization in LPS‐stimulated microglia. Importantly, OX‐A significantly reduced CD86 expression and increased CD206 levels, as demonstrated by both FCM and IF analyses, suggesting that OX‐A promotes the M1 to M2 phenotypic shift in microglia. However, these effects were antagonized by PapRIV treatment. Subsequently, we examined the NF‐κB signaling pathway in BV2 cells.

In line with the FCM and IF data, LPS exposure significantly increased p65 nuclear translocation (Figure 6O,P) and the expression of p‐p65, p‐IκBα, and iNOS (Figure 6Q–U, *p* < 0.001), while Arg‐1 remained unchanged (*p* > 0.05), collectively indicating activation of the NF‐κB signaling pathway in microglia. Conversely, OX‐A effectively reversed LPS‐induced NF‐κB activation and enhanced Arg‐1 expression; notably, these effects were abolished by PapRIV. Taken together, these findings demonstrate that OX‐A attenuates M1 activation and promotes M2 polarization in microglia, thereby mediating anti‐neuroinflammatory effects, potentially through regulation of the NF‐κB signaling pathway.

### 3.7 OX‐A Inhibited Activated Microglia‐Mediated Neuronal Death

**FIGURE 7:**
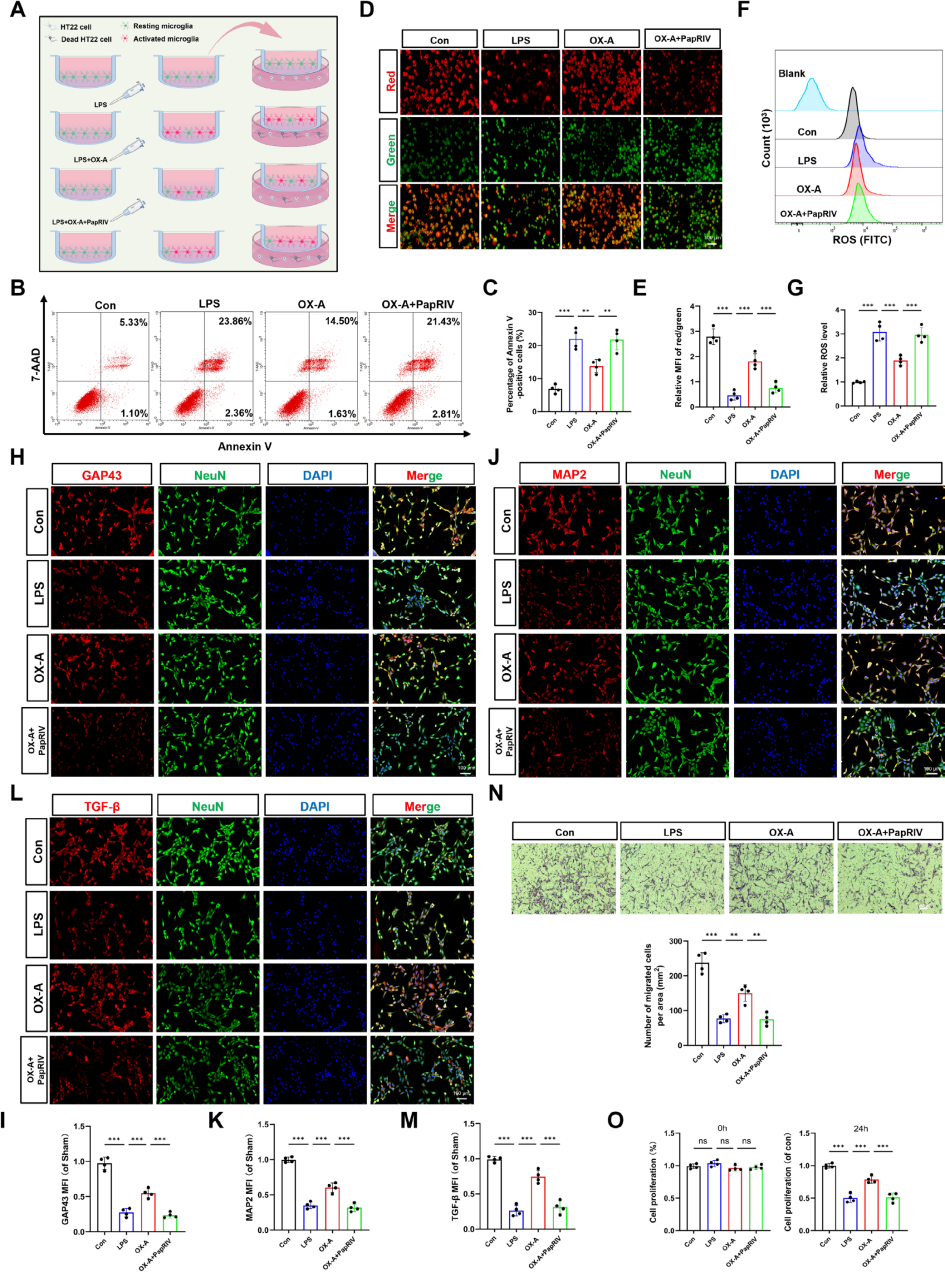
OX‐A inhibited activated microglia‐mediated neuronal death and promoted proliferation and migration. (A) The schematic diagram illustrates the co‐culture of HT22 and BV2 cells. (B, C) Representative FCM images and quantification of 7‐AAD and Annexin V positive cells (*n* = 4 independent measures). (D) Representative images of JC‐1 staining. (E) Quantitative of the red/green ratio in HT22 cells (*n* = 4 independent measures). (F) Representative histograms depicting intracellular ROS level. (G) MFI of ROS was quantified for each group (*n* = 4 independent measures). (H, I) Representative IF images and quantitative analysis of GAP43 and NeuN (*n* = 4 in each group). (J, K) Representative IF images and quantitative analysis of MAP2 and NeuN (*n* = 4 independent measures). (L, M) Representative IF images and quantitative analysis of TGF‐β and NeuN (*n* = 4 independent measures). (N) Representative images and quantification of migrated HT22 cells (*n* = 4 independent measures). (O) Cell proliferation capacity was evaluated using the CCK8 assay at 0 h and 24 h post co‐culture (*n* = 4 independent measures). All values are mean ± SD. Scale = 100 μm. *p < 0.05, **p < 0.01, ***p < 0.001.

To investigate the neuroprotective potential of OX‐A under inflammatory conditions, we established a Transwell coculture system consisting of BV2 microglia and HT22 neurons (Figure 7A). We then assessed the effect of OX‐A on neuronal apoptosis using 7‐AAD/Annexin V staining. Our results indicated that OX‐A significantly reduced microglia‐induced neuronal apoptosis (*p* < 0.01), and this antiapoptotic effect was reversed by PapRIV treatment (Figure 7B,C, *p* < 0.01).

Given that aberrant ROS accumulation in mitochondria can disrupt mitochondrial homeostasis and trigger neuronal apoptosis [46], we examined ROS levels and MMP. Our findings revealed that LPS treatment significantly elevated intracellular ROS levels while reducing MMP in HT22 cells compared to the Con group (*p* < 0.001). OX‐A administration effectively counteracted these effects, substantially decreasing ROS generation and restoring MMP (*p* < 0.001), indicating its capacity to maintain mitochondrial function by inhibiting ROS. However, these beneficial effects were substantially attenuated following PapRIV treatment (Figure 7D–G).

To further elucidate the neuroprotective mechanisms of OX‐A, we investigated its effects on neural repair and proliferation markers in HT22 cells following coculture with microglia. Specifically, we assessed the expression of MAP2, GAP43, and TGF‐β, along with cell proliferation and migration capabilities. Our findings revealed that coculturing with OX‐A‐pretreated microglia significantly upregulated the expression of MAP2, GAP43, and TGF‐β proteins in HT22 cells compared to the LPS group (Figure 7H–M, *p* < 0.001). Furthermore, OX‐A pretreatment enhanced HT22 cell proliferation (*p* < 0.01) and improved cell migration (Figure 7N,O, *p* < 0.01). Notably, these OX‐A‐mediated effects were reversed by PapRIV treatment. Taken together, these results indicate that OX‐A exerts neuroprotective effects by modulating the OX1R‐mediated NF‐κB pathway, which leads to diminished neuroinflammatory responses, decreased neuronal apoptosis, and increased neuronal proliferation and migration capabilities.

## 4 Discussion

TBI is a severe neurological condition resulting in a range of neurological impairments. Traditional methods of TBI rehabilitation, however, have shown limited effectiveness [50]. In this study, we observed that TBI exposure induced neuroinflammation and BBB disruption in the cortex and hippocampus, accompanied by neurodegenerative changes and motor and cognitive dysfunction. Subsequently, we found that tPCS ameliorated microglia‐mediated neuroinflammation and neurological deficit, potentially via activation of the OX‐A/OX1R pathway. Further in vitro experiments demonstrated that OX‐A suppressed LPS‐activated microglial NF‐κB signaling, reducing M1 polarization and promoting a shift toward the M2 phenotype. These findings provide compelling evidence that tPCS represents a promising neuromodulation technique for promoting neurorehabilitation in TBI patients.

Accumulating clinical evidence highlights the potential of tPCS as a noninvasive neuromodulation technique to modulate the excitability of both cortical and subcortical structures [51], improving motor function [21], and enhancing cognitive abilities [20]. Emerging research also suggests tPCS possesses notable anti‐inflammatory and neurorestorative capabilities, specifically upregulating neurotrophic factors expression [23] and reducing pro‐inflammatory cytokines in the peri‐infarct region [24], thereby mitigating secondary damage and neurological deficits, although the precise mechanisms remain largely unknown. In this study, we observed that TBI‐induced multiple pathological changes in the peri‐lesional cortex, including neuroinflammation, neurodegenerative alterations, and diminished neuroplasticity, which correlated with neurological dysfunction. Notably, our findings demonstrate that tPCS augmented neurotrophic factor pathways and TGF‐β signaling. Further investigation revealed that tPCS significantly upregulated OX‐A expression and inhibited M1 microglia‐mediated neuroinflammation. Concurrently, tPCS enhanced the expression of neurorepair‐associated markers and neuroplasticity, ultimately ameliorating neurological deficits following TBI. However, these beneficial effects were abolished by the OX1R antagonist SB334867. These findings strongly suggest that tPCS‐mediated neuroprotection may be achieved through activation of the lateral hypothalamus, subsequently increasing neuropeptide OX‐A secretion.

Neurorepair is intricately regulated by various neurotrophic factors and regulatory proteins, with MAP2, GAP43, and TGF‐β recognized as key players [46]. MAP2, a fundamental cytoskeletal protein localized in neuronal somas and dendrites, is crucial for microtubule assembly and stabilization, dendritic extension, and neuronal structure maintenance [52]. Similarly, GAP43 and TGF‐β, growth‐associated proteins widely expressed in the CNS, are critical mediators in neural development and repair [53]. Extensive research indicates that the expression levels of these proteins correlate with neurorepair and neuroplasticity in central nervous system injuries, including traumatic brain injury, spinal cord injury, and ischemic stroke [46, 54, 55]. In this study, our data thoroughly evaluated the protein levels of MAP2, GAP43, and TGF‐β within the peri‐lesional cortex. Our findings revealed a notable decrease in the expression of these proteins at 7 dpo, contrasting with previous research [23]. We postulate that this discrepancy might be attributed to variations in the disease model employed, injury severity, and assessment time points. Furthermore, our observations aligned with existing literature in demonstrating reduced neuronal morphological complexity and diminished synaptic plasticity of TBI mice [56]. Notably, tPCS treatment enhances the expression of neurorepair and synaptic plasticity markers. However, SB334867 blocked these benefits, suggesting that tPCS‐induced neuroprotection is partially dependent on OX‐A/OX1R pathway activation.

Microglia, exhibiting remarkable plasticity, are commonly categorized into M1 and M2 phenotypes, representing the opposite ends of their activation spectrum [6]. Although this binary classification remains debated, it provides a valuable framework for understanding microglial function in various neuroinflammatory conditions, including stroke and TBI [57, 58]. In the acute phase of TBI, M1 microglia‐mediated neuroinflammation significantly contributes to secondary injury mechanisms. Consistent with this, studies have demonstrated that promoting the shift from M1 to M2 polarization can effectively mitigate neuroinflammation and improve neurological recovery [8]. In line with these findings, our research revealed a predominant M1 microglial activation in the perilesional cortex of TBI mice, which correlated with increased neuroinflammation and apoptosis. Notably, tPCS treatment attenuated these pathological processes by promoting M2 microglial polarization, increasing anti‐inflammatory cytokine levels, and upregulating neurorepair‐associated proteins. These findings underscore the critical role of microglia in TBI pathophysiology and highlight the therapeutic potential of tPCS in restoring microglial homeostasis and promoting anti‐inflammatory and neuroreparative effects.

Mounting evidence indicates that the NF‐κB pathway plays a crucial role in mediating the post‐TBI inflammatory cascade [43]. During injury, various factors, including hypoxia, ROS, and pro‐inflammatory mediators, trigger the activation and nuclear translocation of the NF‐κB/p65 subunit, initiating the transcription of downstream genes and exacerbating neuroinflammation [59]. This suggests that targeting NF‐κB signaling may be a promising therapeutic strategy for mitigating secondary damage after TBI. OX‐A, a novel anti‐neuroinflammatory peptide, exhibits significant neuroprotective properties. Prior research, including our own, has established that OX‐A mitigates neuronal injury by inhibiting LPS‐induced NF‐κB activation [29]. Consistent with these findings, intraventricular administration of OX‐A has been shown to effectively suppress NF‐κB‐mediated neuroinflammation and improve neurological outcomes following stroke [60]. Furthermore, OX‐A can modulate macrophage polarization, alleviating poststroke immunosuppression [45]. In the present study, we observed a reduction in OX‐A expression following both TBI and LPS exposure, which coincided with enhanced NF‐κB pathway activation and M1 microglial polarization. Notably, tPCS upregulated OX‐A expression and concurrently suppressed NF‐κB/p65 activation and M1 microglial polarization. This effect of tPCS was significantly attenuated by the administration of SB334867. Supporting these in vivo observations, in vitro experiments demonstrated that OX‐A inhibited LPS‐induced NF‐κB activation, promoted microglial transition from the M1 to the M2 phenotype, reduced neuronal apoptosis, and enhanced cell proliferation and migration. Importantly, these neuroprotective effects were reversed by PapRIV. These findings collectively suggest that the OX‐A/OX1R/NF‐κB pathway is critical for TPCS's ability to suppress neuroinflammation and promote neurological recovery.

Several limitations warrant consideration in this study. First, our investigation focused on the effects of tPCS and OX‐A on microglial function and their subsequent enhancement of neurorepair‐related markers and neuroplasticity. However, findings suggest tPCS may also promote the proliferation and signaling of astrocytes, endothelial cells, and potentially other cell types. Whether these neuroprotective and nonspecific effects are parameter‐dependent and contribute to neurofunctional integration after TBI requires further investigation. Additionally, our findings indicate that tPCS enhances OX‐A/OX1R signaling, providing neuroprotection without adverse effects. However, previous research suggests that excessive OX‐A signaling activation could lead to neuronal hyperexcitability, potentially increasing the risk of insomnia and seizures. Future studies should systematically evaluate various stimulation parameters to identify safer and more effective protocols. Finally, while this study assessed the impact of tPCS on neurorepair and neuroplasticity in the acute phase, direct evidence of functional integration of newly generated neurons was lacking. Future research should therefore investigate the long‐term functional integration and lasting effects of these new neurons through extended monitoring.

In conclusion, we demonstrated that tPCS effectively restores microglial homeostasis after TBI by modulating the OX‐A/OX1R‐mediated NF‐κB pathway. This modulation subsequently reduces neuroinflammation, enhances neuroplasticity, and improves neurological function. These findings provide strong preclinical evidence for the therapeutic potential of tPCS in neurological rehabilitation after TBI.

## Author Contributions

Zhen Feng and Yang Bai designed and supervised the study. Bingkai Ren and Qianhui Zhou performed experiments. Peng Yao analyzed the data and wrote the paper. All authors read and approved the final work.

## Ethics Statement

This study's animal experiments were approved by the Animal Care and Use Committee of Nanchang University (ID: CDYFY‐IACUC‐202302QR058). These experiments were performed in strict accordance with the ethical standards established by the National Institutes of Health for the care and use of laboratory animals. Additionally, all methodologies employed in the study are documented in compliance with the ARRIVE guidelines for reporting animal experiments.

## Conflicts of Interest

The authors declare no conflicts of interest.

## Supporting information


**Figure S1:** The impact of tPCS on the expression of OX2R in the peri‐lesional cortex of TBI mice. (A, B) Representative IF images and quantification depict the expression levels of OX2R at 7 dpo (*n* = 4). (C, D) Representative Western blot images and quantification illustrate the levels of OX2R in the peri‐lesional cortex (*n* = 4), with β‐actin serving as the loading control. The original blots are presented in Appendix S1. All values are mean ± SD. *p < 0.05, **p < 0.01, ***p < 0.001.
**Figure S2:** The impact of tPCS on the histopathology of the hippocampus and cognitive function in TBI mice. (A–F) Cognitive function in TBI mice was assessed using the Morris water maze and novel object recognition tests. Metrics included escape latency, time spent in the target quadrant, swim speed, and the recognition index (*n* = 8). White circles indicate the novel object in MWM. (G) Representative images of the hippocampus were obtained using H&E and Nissl staining. (H) Quantification of Nissl‐stained cells (*n* = 4). (I, J) Representative immunohistochemical staining and quantitative analysis of OXA (*n* = 4). (K, L) Representative TUNEL staining and quantitative analysis (*n* = 4). (M–O) Representative IF images and quantitative analysis of Bcl‐xL and NeuN (*n* = 4). (P, Q) Representative IF images and quantitative analysis of TGF‐β and NeuN (*n* = 4). (R–V) Representative Western blot bands and quantitative analysis demonstrating Bcl‐xL, NeuN, OX‐A and OX1R levels in the hippocampus (*n* = 3), with β‐actin employed as the loading control. The original blots are presented in Appendix S1. (W–X) Representative IF images and quantification of NeuN+BrdU‐positive cells in the hippocampus (*n* = 4). The arrows represent NeuN+BrdU‐positive cells. Scale bar = 100 μm. (Y‐Z) Representative IF images and quantification of CD31+BrdU‐positive cells (*n* = 4). The arrows represent CD31+BrdU positive cells. Scale bar = 100 μm. All values are mean ± SD. *p < 0.05, **p < 0.01, ***p < 0.001.
**Figure S3:** The impact of tPCS on neuroregeneration and angiogenesis in the contralateral cortex following TBI. (A) Representative IF images of NeuN+BrdU in the contralateral cortex at 7 dpo (*n* = 6). (B, C) Quantification of NeuN+BrdU‐positive cells within the contralateral cortex, alongside the ratio of these cells in the injured cortex to the contralateral cortex (*n* = 6). (D) Representative IF images of CD31+BrdU in the contralateral cortex at 7 dpo (*n* = 6). (E, F) Quantification of CD31+BrdU‐positive cells, alongside the ratio of these cells in the injured cortex to the contralateral cortex (*n* = 6). Scale bar = 100 μm. All values are mean ± SD. *p < 0.05, **p < 0.01, ***p < 0.001.
**Figure S4:** The impact of tPCS on the proliferation of astrocytes and microglial cells in the peri‐lesional cortex following TBI. (A) Representative IF images of GFAP+BrdU in the peri‐lesional cortex at 7 dpo (*n* = 6). (B, C) Quantification of GFAP+BrdU‐positive cells (*n* = 6). (D) Representative IF images of IBA1+BrdU in the peri‐lesional cortex at 7 dpo (*n* = 6). (E, F) Quantification of IBA1+BrdU‐positive cells (*n* = 6). Scale bar = 100 μm. All values are mean ± SD. *p < 0.05, **p < 0.01, ***p < 0.001.
**Figure S5:** The impact of tPCS on the polarization of microglial cells in the contralateral cortex following TBI. (A) Representative IF images of IBA1+CD68 in the contralateral cortex at 7 dpo (*n* = 6). (B, C) Quantification of IBA1+CD68‐positive cells in the contralateral cortex, alongside the ratio of these cells in the injured cortex to the contralateral cortex (*n* = 6). (D) Representative IF images of IBA1+CD206 in the contralateral cortex at 7 dpo (*n* = 6). (E, F) Quantification of IBA1+CD206‐positive cells, alongside the ratio of these cells in the injured cortex to the contralateral cortex (*n* = 6). Scale bar = 100 μm. All values are mean ± SD. *p < 0.05, **p < 0.01, ***p < 0.001.

**Appendix S1:** cns70606‐sup‐0002‐AppendixS1.pdf.

## Data Availability

The data presented in this study are available on reasonable request from the corresponding author (fengzhen@email.ncu.edu.cn).
