# Supplementary figures and images for: Neuroprotective Effects of Transcranial Pulsed Current Stimulation: Modulation of Microglial Polarization in Traumatic Brain Injury
Source: CNS Neurosci Ther. 2025 Sep 7;31(9):e70606. doi: 10.1111/cns.70606 (PMC12415356; doi:10.1111/cns.70606)

**Fig.S1**

**
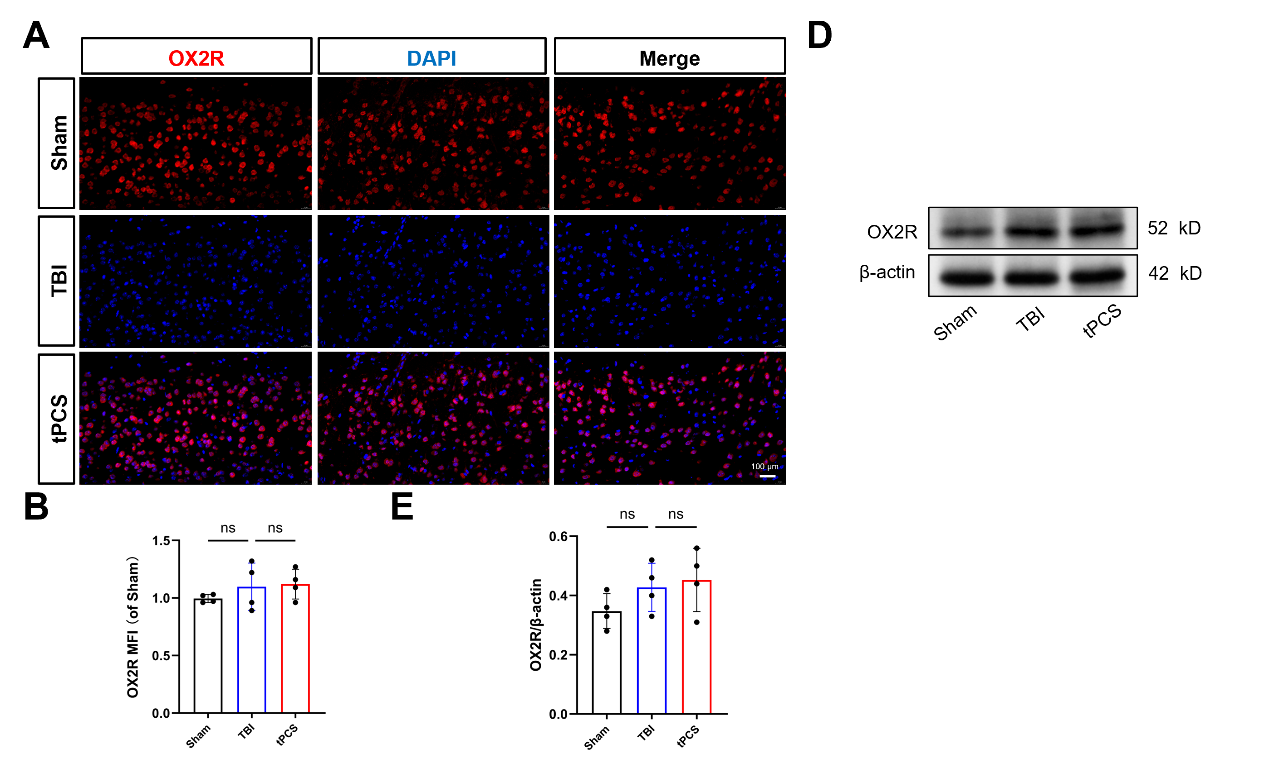
**

**Fig.S2**


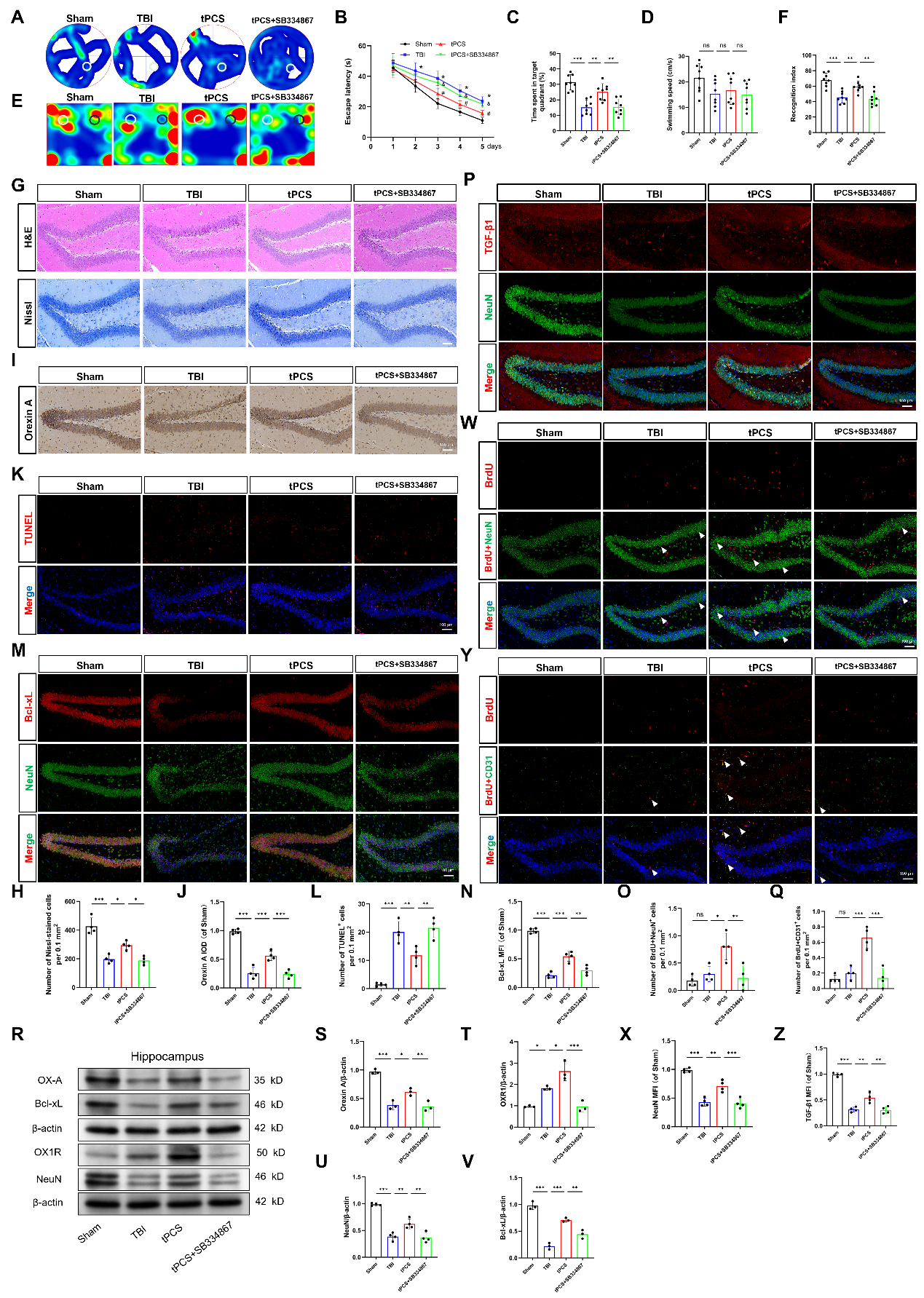


**Fig.S3**


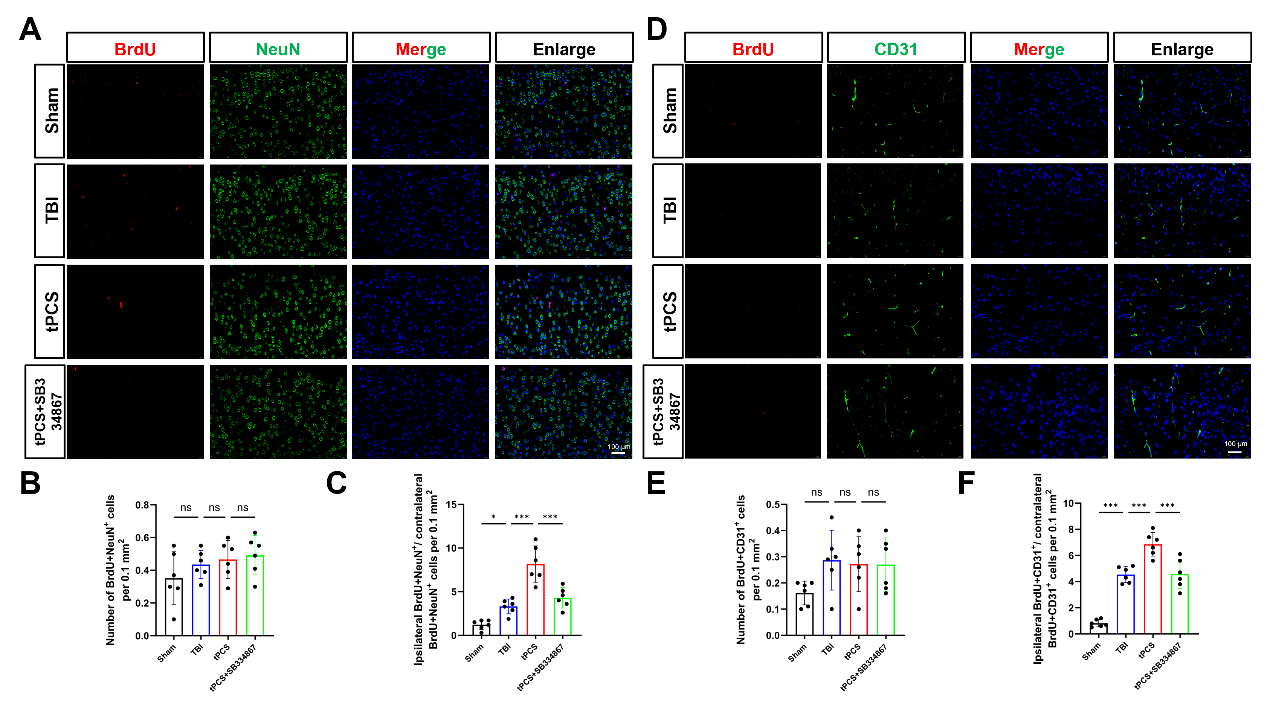


**Fig.S4
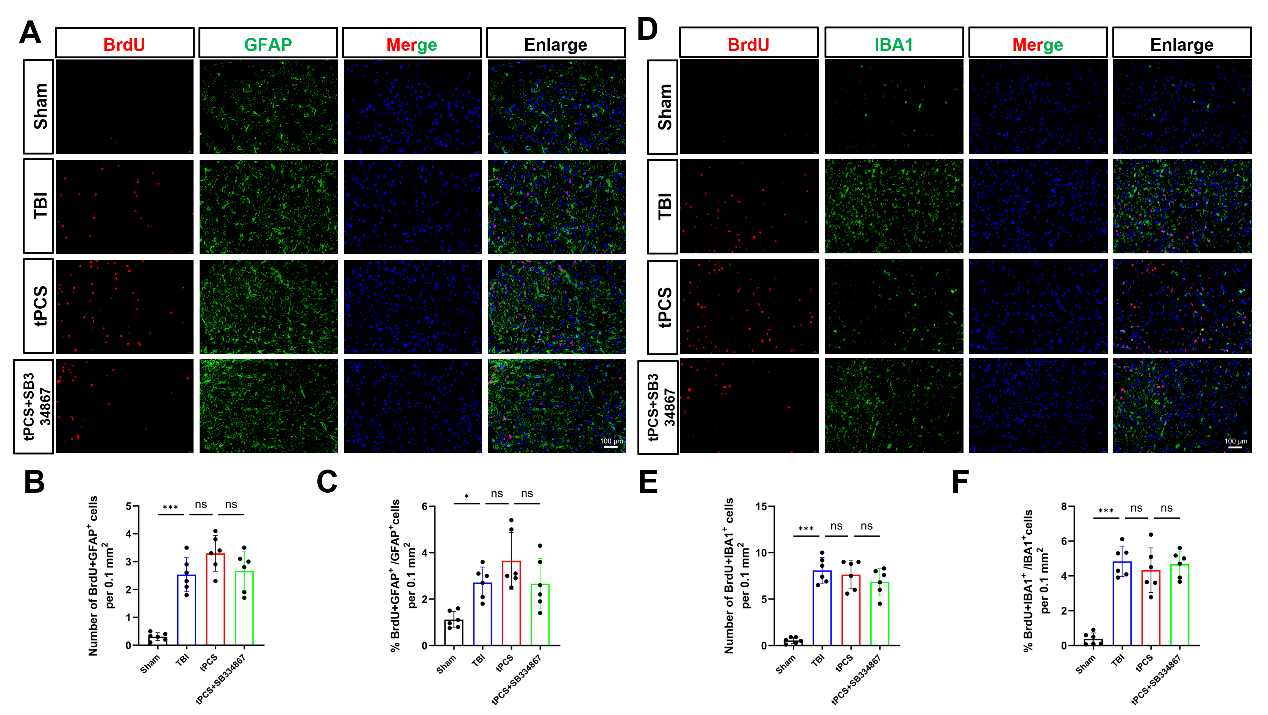
**

**Fig.S5**


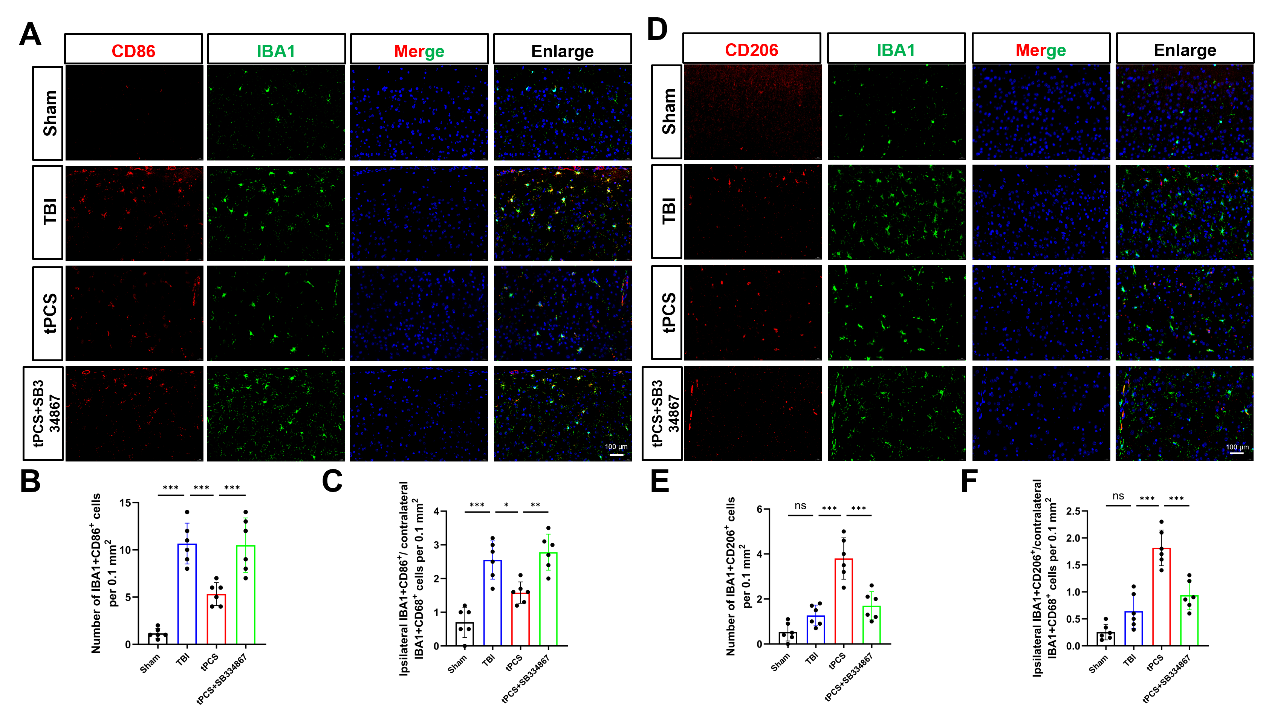

Supplement: Supplementary file 1 — Figure S1: The impact of tPCS on the expression of OX2R in the peri‐lesional cortex of TBI mice. (A, B) Representative IF images and quantification depict the expression levels of OX2R at 7 dpo (n = 4). (C, D) Representative Western blot images and quantification illustrate the levels of OX2R in the peri‐lesional cortex (n = 4), with β‐actin serving as the loading control. The original blots are presented in Appendix S1. All values are mean ± SD. *p < 0.05, **p < 0.01, ***p < 0.001. Figure S2: The impact of tPCS on the histopathology of the hippocampus and cognitive function in TBI mice. (A–F) Cognitive function in TBI mice was assessed using the Morris water maze and novel object recognition tests. Metrics included escape latency, time spent in the target quadrant, swim speed, and the recognition index (n = 8). White circles indicate the novel object in MWM. (G) Representative images of the hippocampus were obtained using H&E and Nissl staining. (H) Quantification of Nissl‐stained cells (n = 4). (I, J) Representative immunohistochemical staining and quantitative analysis of OXA (n = 4). (K, L) Representative TUNEL staining and quantitative analysis (n = 4). (M–O) Representative IF images and quantitative analysis of Bcl‐xL and NeuN (n = 4). (P, Q) Representative IF images and quantitative analysis of TGF‐β and NeuN (n = 4). (R–V) Representative Western blot bands and quantitative analysis demonstrating Bcl‐xL, NeuN, OX‐A and OX1R levels in the hippocampus (n = 3), with β‐actin employed as the loading control. The original blots are presented in Appendix S1. (W–X) Representative IF images and quantification of NeuN+BrdU‐positive cells in the hippocampus (n = 4). The arrows represent NeuN+BrdU‐positive cells. Scale bar = 100 μm. (Y‐Z) Representative IF images and quantification of CD31+BrdU‐positive cells (n = 4). The arrows represent CD31+BrdU positive cells. Scale bar = 100 μm. All values are mean ± SD. *p < 0.05, **p < 0.01, ***p < 0.001. Figure S3: The impact of [file CNS-31-e70606-s002.docx]
