# Supplementary material for: Neuroprotective Effects of Transcranial Pulsed Current Stimulation: Modulation of Microglial Polarization in Traumatic Brain Injury
Source: CNS Neurosci Ther. 2025 Sep 7;31(9):e70606. doi: 10.1111/cns.70606 (PMC12415356; doi:10.1111/cns.70606)

StarSingal Western Protein Marker (10-200 kDa) ,

cat. ZM227-101, GenStar, China https://www.gene-star.com/product_view.php?id=678

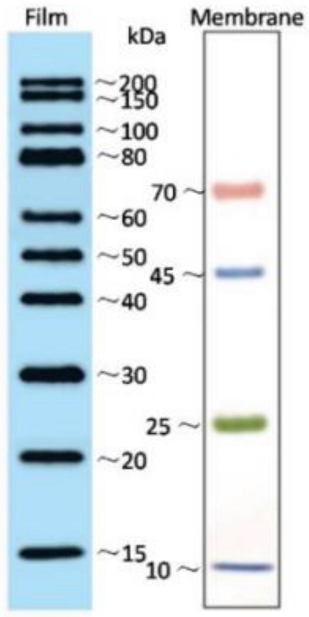

Full unedited blot for Figure 2

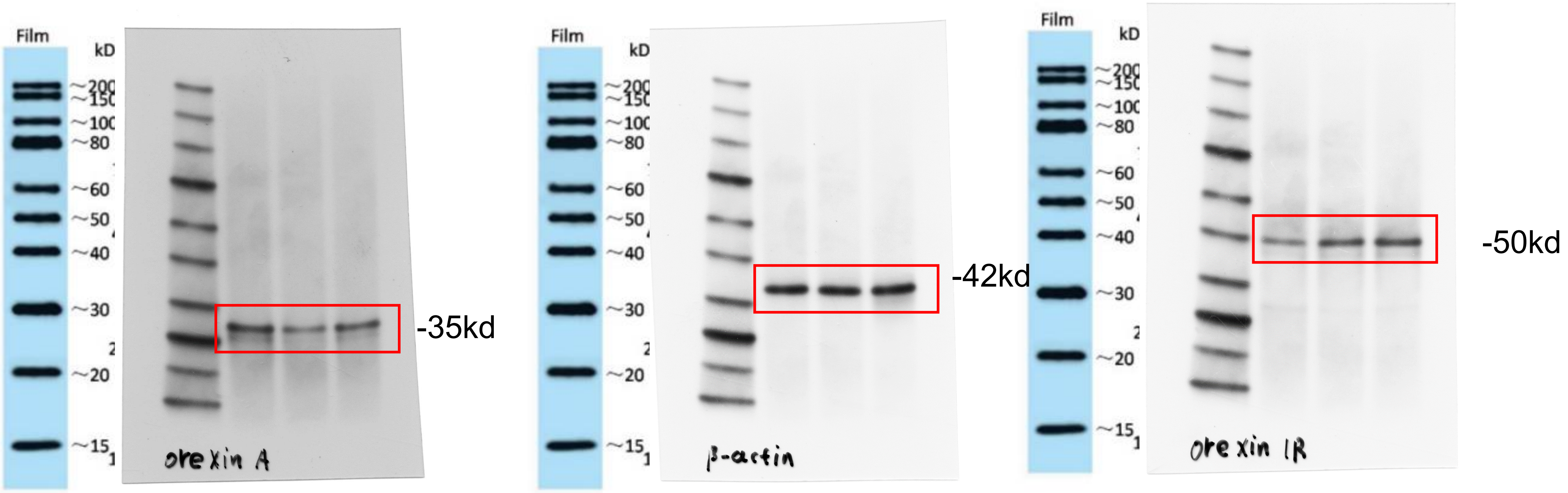

Full unedited blot for Figure 2

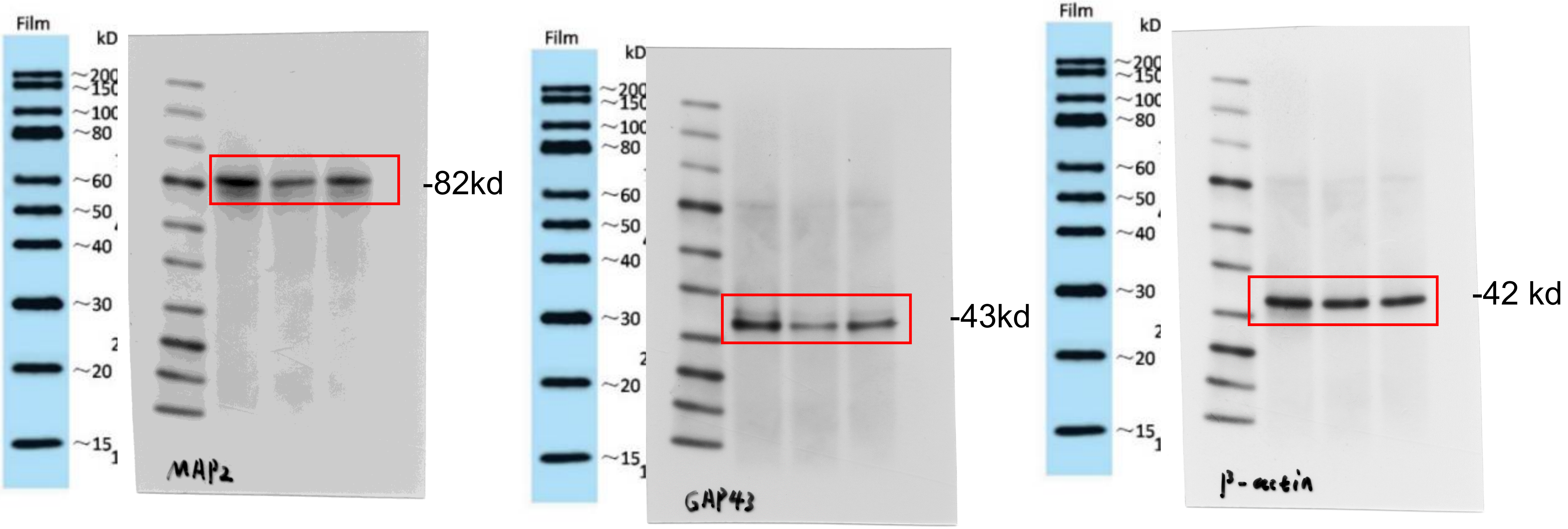

Full unedited blot for Figure 3

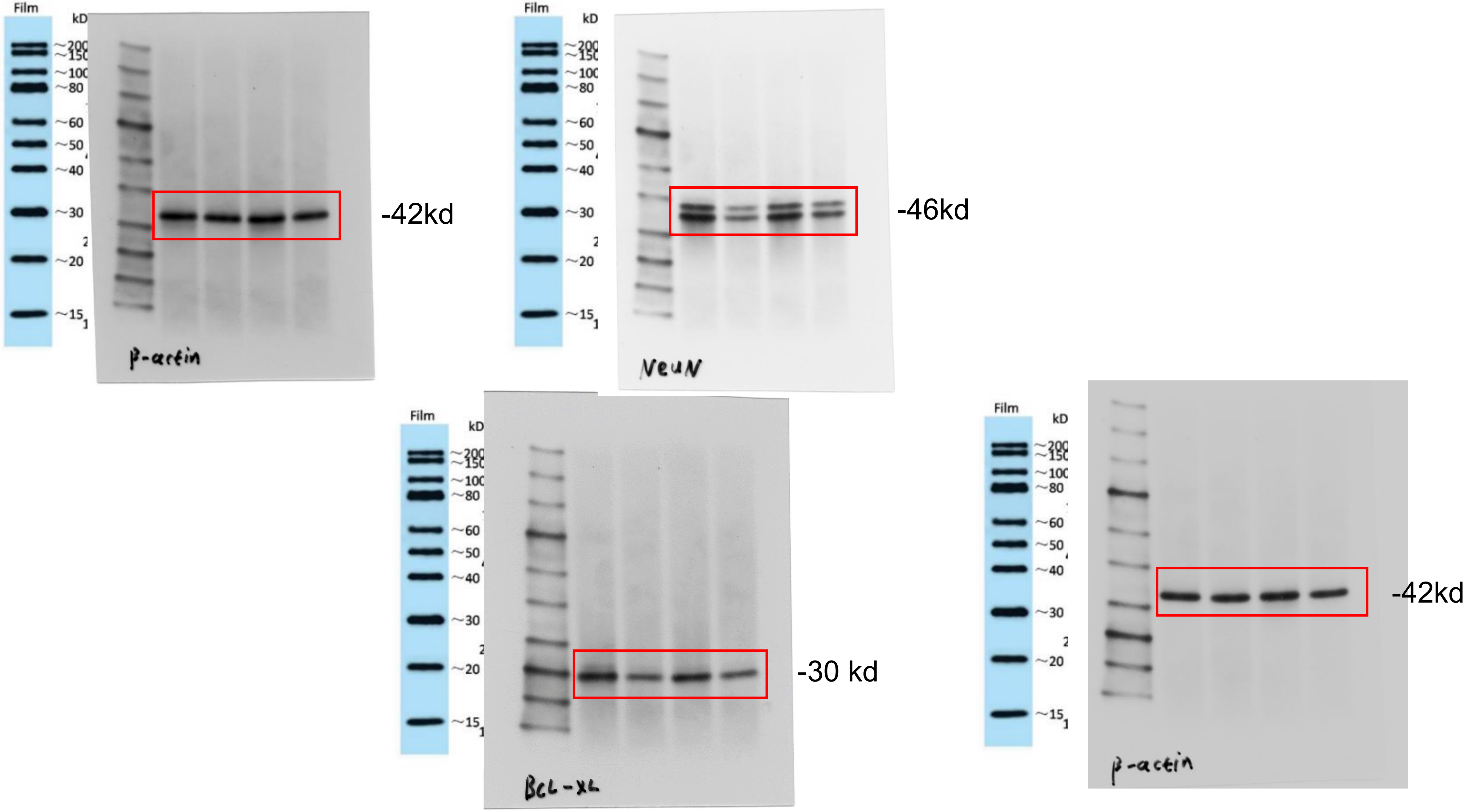

Full unedited blot for Figure 4

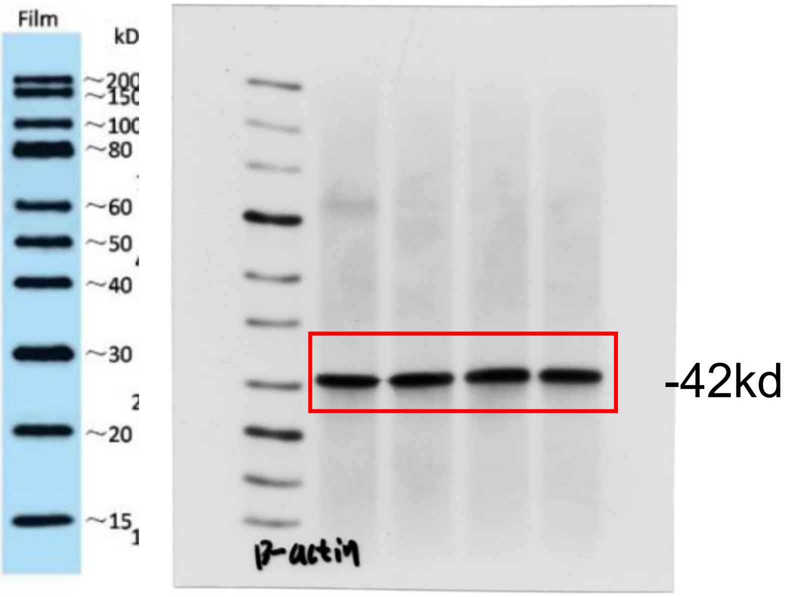

-42kd

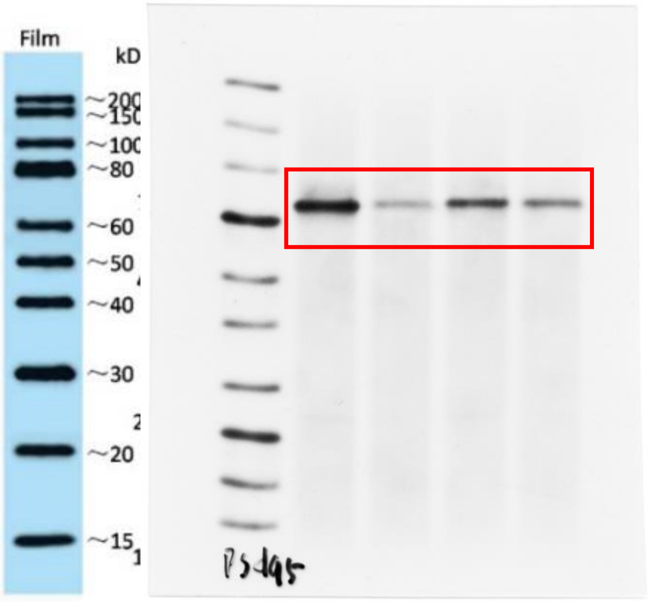

-85kd

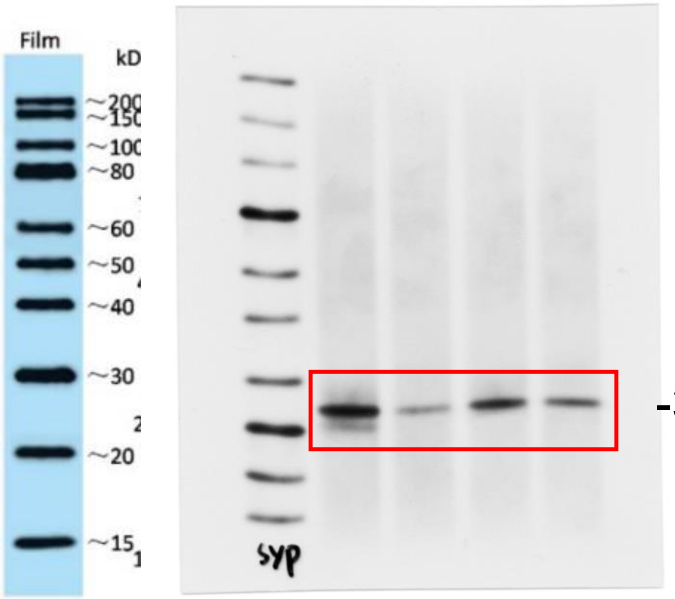

-34kd

Full unedited blot for Figure 5

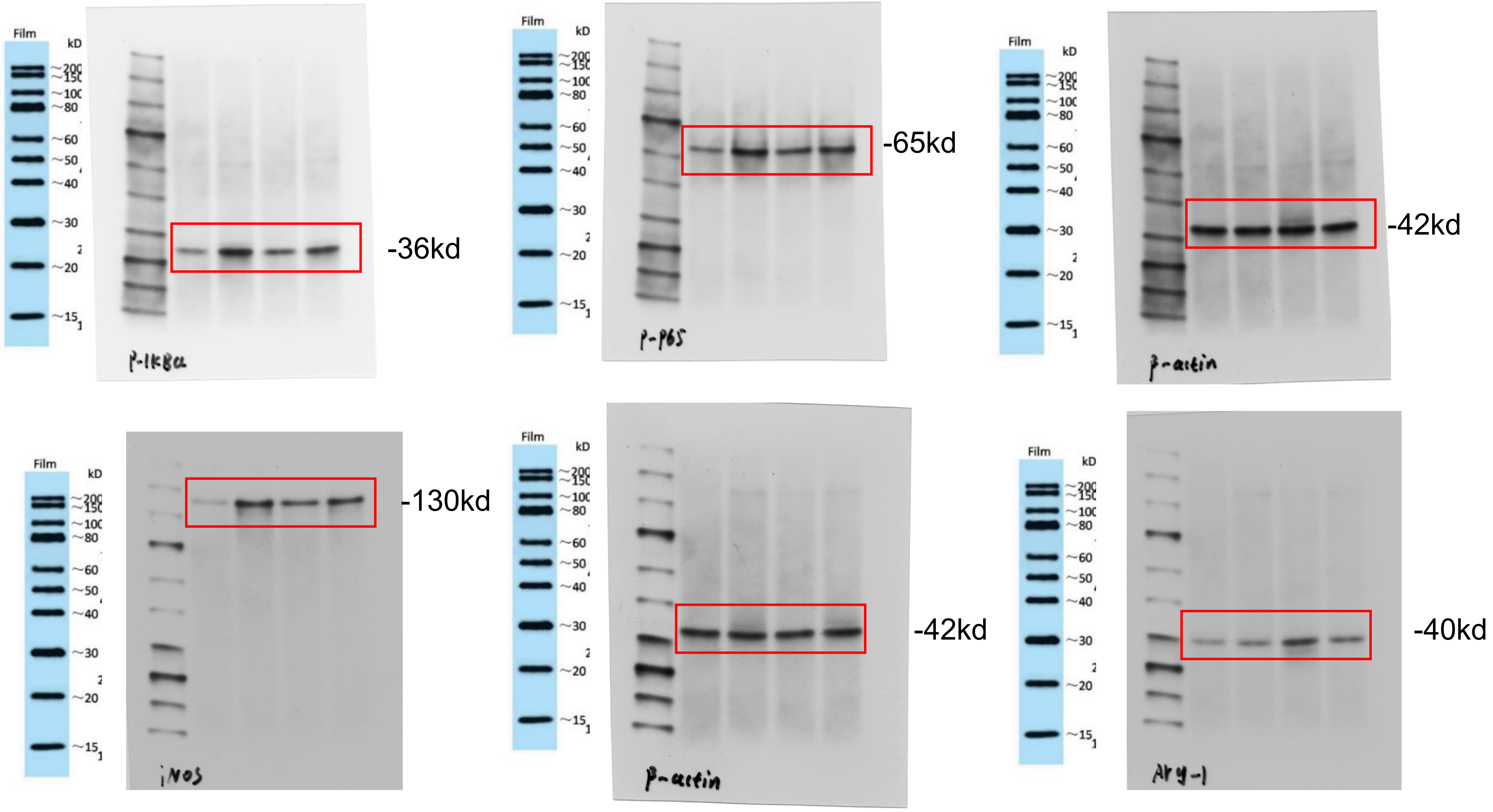

Full unedited blot for Figure 6

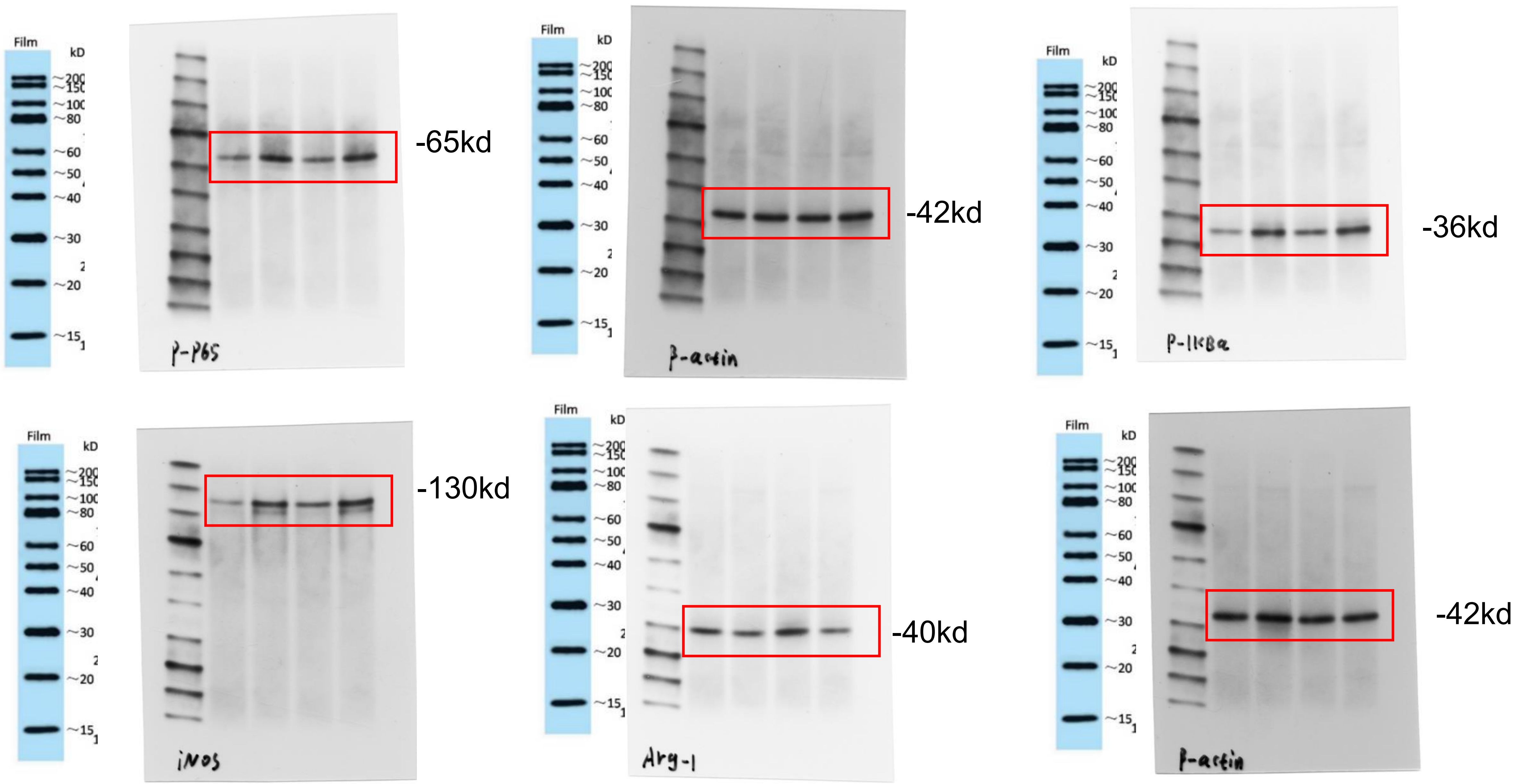

Full unedited blot for Figure S1

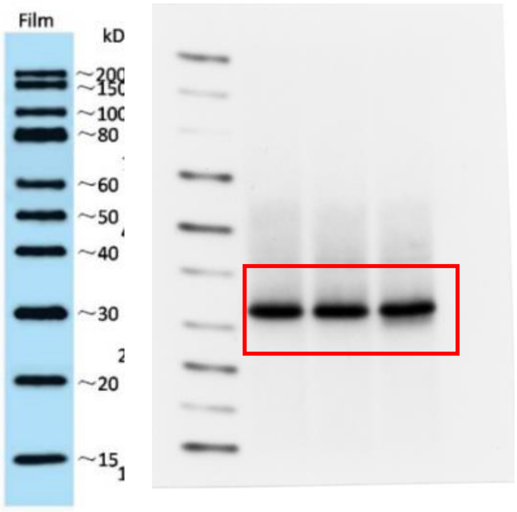

-42kd

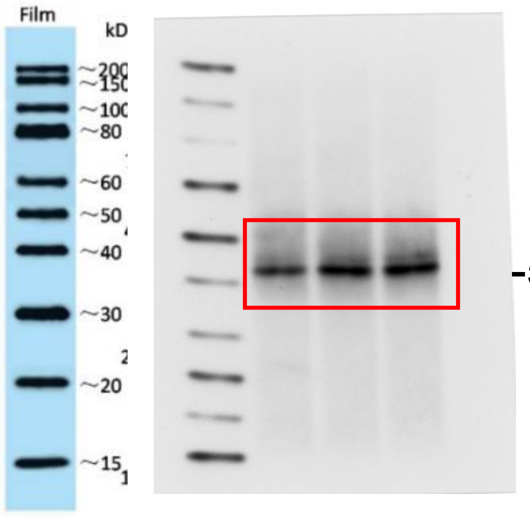

-52kd

Full unedited blot for Figure S2

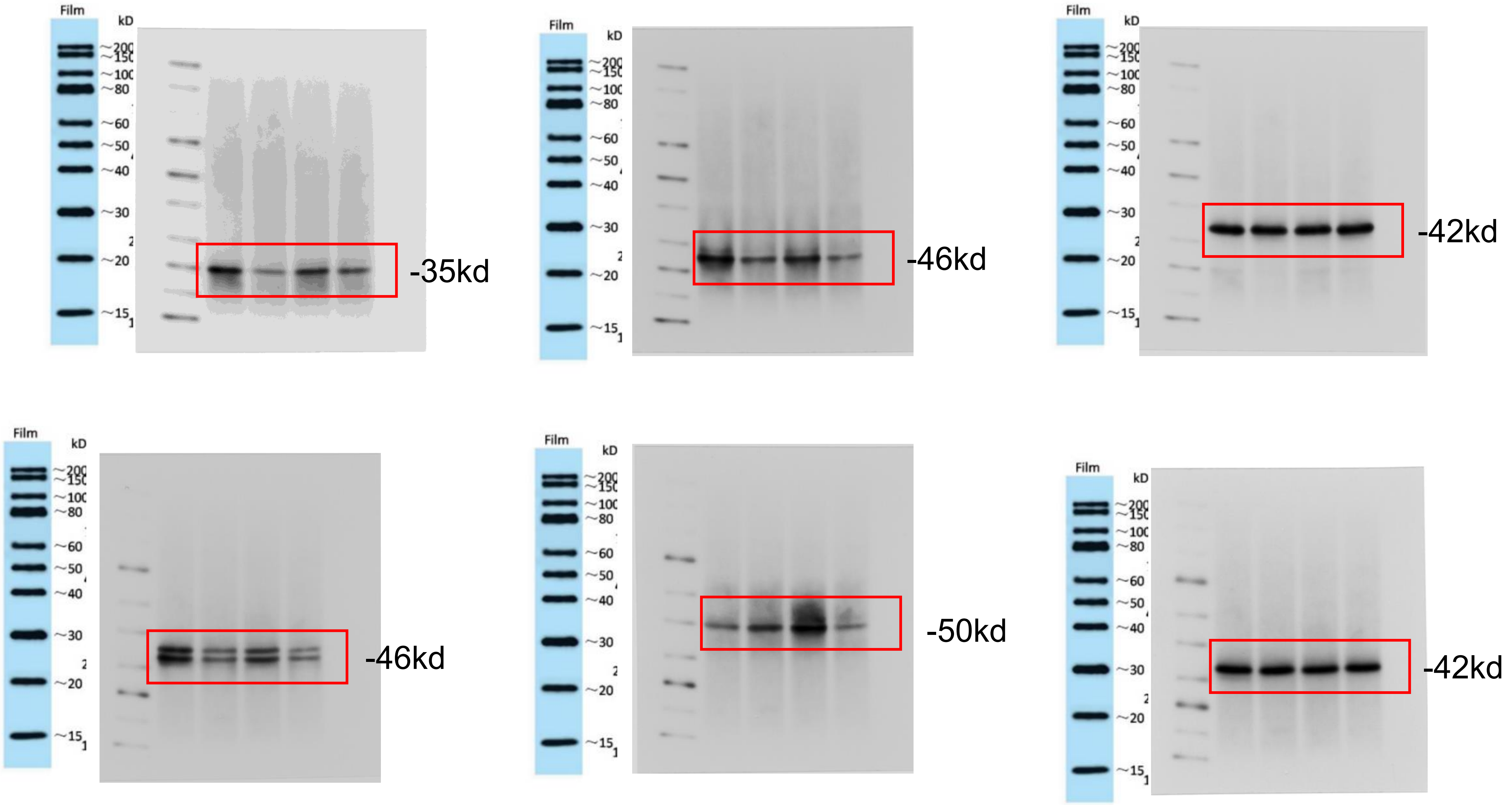

Supplement: Supplementary file 2 — Appendix S1: cns70606‐sup‐0002‐AppendixS1.pdf. [file CNS-31-e70606-s001.pdf]
